# “The person in power told me to”—European PhD students’ perspectives on guest authorship and good authorship practice

**DOI:** 10.1371/journal.pone.0280018

**Published:** 2023-01-12

**Authors:** Mads Paludan Goddiksen, Mikkel Willum Johansen, Anna Catharina Armond, Christine Clavien, Linda Hogan, Nóra Kovács, Marcus Tang Merit, I. Anna S. Olsson, Una Quinn, Júlio Borlido Santos, Rita Santos, Céline Schöpfer, Orsolya Varga, P. J. Wall, Peter Sandøe, Thomas Bøker Lund

**Affiliations:** 1 Department of Food and Resource Economics, University of Copenhagen, Copenhagen, Denmark; 2 Department of Science Education, University of Copenhagen, Copenhagen, Denmark; 3 Centre for Journalology, Ottawa Hospital Research Institute, Ottawa, Canada; 4 Department of Public Health and Epidemiology, University of Debrecen, Debrecen, Hungary; 5 Institut Éthique Histoire Humanités, Université de Genève, Geneva, Switzerland; 6 School of Religion, Trinity College Dublin, Dublin, Ireland; 7 Institute of Architecture, Urbanism and Landscape, Royal Danish Academy, Copenhagen, Denmark; 8 i3S – Instituto de Investigação e Inovação em Saúde, Universidade do Porto, Porto, Portugal; 9 School of Ecumenics, Trinity College Dublin, Dublin, Ireland; 10 ADAPT Centre, Trinity College Dublin, Dublin, Ireland; 11 Department of Veterinary and Animal Sciences, University of Copenhagen, Copenhagen, Denmark; University of Texas Southwestern Medical Center at Dallas, UNITED STATES

## Abstract

Questionable authorship practices in scientific publishing are detrimental to research quality and management. The existing literature dealing with the prevalence, and perceptions, of such practices has focused on the medical sciences, and on experienced researchers. In contrast, this study investigated how younger researchers (PhD students) from across the faculties view fair authorship attribution, their experience with granting guest authorships to more powerful researchers and their reasons for doing so. Data for the study were collected in a survey of European PhD students. The final dataset included 1,336 participants from five European countries (Denmark, Hungary, Ireland, Portugal, and Switzerland) representing all major disciplines. Approximately three in ten reported that they had granted at least one guest authorship to “a person in power”. Half of these indicated that they had done so because they had been told to do so by the person in power. Participants from the medical, natural and technical sciences were much more likely to state that they had granted a guest authorship than those from other faculties. We identified four general views about what is sufficient for co-authorship. There were two dominant views. The first (inclusive view) considered a broad range of contributions to merit co-authorship. The second (strongly writing-oriented) emphasised that co-authors must have written a piece of the manuscript text. The inclusive view dominated in the natural, technical, and medical sciences. Participants from other faculties were more evenly distributed between the inclusive and writing oriented view. Those with an inclusive view were most likely to indicate that they have granted a guest authorship. According to the experiences of our participants, questionable authorship practices are prevalent among early-career researchers, and they appear to be reinforced through a combination of coercive power relations and dominant norms in some research cultures, particularly in the natural, technical, and medical sciences.

## 1 Introduction

To build a research career it is essential to author or co-author influential research publications. The criteria for legitimate co-authorship are therefore widely discussed, and questionable authorship practices are often pointed to as a major problem for research integrity [1–3]. Most questionable authorship practices fall short of legal definitions of research misconduct, which typically include only falsification, fabrication and plagiarism. However, they are still detrimental to the trustworthiness of research, and to the fair competition among researchers for funding and positions. Additionally, they can be detrimental to the careers and wellbeing of the individual researchers who can feel exploited either because they do not get the an authorship they feel they deserve or because they see authorships being granted for much less than what is required of them. The most common questionable authorship practice seems to be *guest* authorship, where people who have not made a significant contribution to the study in question are made co-authors. This is also sometimes referred to as “honorary authorship” or “gift authorship”. Guest authorships are problematic. Among other things, they provide guest authors with an undeserved share of the credit for the study and reduce transparency about who contributed what to the study [4].

Additionally, guest authorships may be a result of unethical ways of collaborating, including coercion of junior researchers by more senior researchers in the sense that a senior researcher instructs a junior researcher that one or more people who did not make a significant contribution to the study should be co-authors–so-called *coerced authorship* [5].

Promoting good authorship practice is thus not only about facilitating fair competition among researchers by ensuring that all and only those who merit a share of the credit for a study are given it. It is also about increasing transparency about who has contributed what to a given piece of research, and promoting fairness in collaborations involving unequal power relations.

Promoting good authorship practice requires knowledge of what it entails, what deviations from the ideal are most common and most harmful, and why the deviations occur.

At an abstract level there is a broad consensus that the central norms of good authorship practice are *transparency* and *responsibility* [6,7]. Fair attribution of authorship involves transparency about who contributed to a study by listing all and only those who made a significant contribution. Additionally, the co-authors of a research publication are responsible for the quality and honesty of its contents–although the precise distribution of this responsibility is sometimes unclear [8].

While the general norms of good authorship practice imply that guest authorship is a questionable practice across the academy, there is no consensus on how to translate the general norms into specific criteria for authorship [9]. The most influential formulation of such criteria is provided by the International Committee of Medical Journal Editors (ICMJE) [10]. According to this, authorship should be granted to all and only those who have made 1) “substantial contributions to the conception or design of the work; or the acquisition, analysis, or interpretation of data for the work” *and* contributed to the 2) drafting or critical revision of the work. In addition, co-authors must have 3) approved the final version of the manuscript, *and* 4) agree “to be accountable for all aspects of the work in ensuring that questions related to the accuracy or integrity of any part of the work are appropriately investigated and resolved” [10].

The ICMJE criteria are–with minor variations [11]–endorsed by many of the most influential journals [12,13] and institutions (e.g. [14]) particularly within the medical and natural sciences. However, it is also widely acknowledged that there are disciplinary differences in the way authorship is assigned.

There are indications that official definitions adopted by journals and institutions only guide daily practice to a limited extent [15]. Instead, precedence and pragmatics seem to guide researchers in decisions about who becomes a co-author [15]. It is therefore important to understand how practicing researchers perceive deserved authorship, and how these perceptions differ across relevant factors like faculty, seniority and country. Unfortunately, the current literature stems largely from medical sciences, and the humanities, and social sciences (except psychology) are largely absent (exceptions include [16–18]). Further, very few studies compare across faculties [19]. A recent exception is the study by Johann & Mayer [20], which found interesting differences across faculties in perceptions of what is sufficient for deserved authorship. The study identified five general views on deserved authorship among senior researchers from across the academy working in Germany. It is worth noting that all five views were more inclusive than the ICMJE criteria (Patience and colleagues [9] found similar results in a study spanning countries across the globe). We discuss these findings in Section 4.

Discrepancies between researchers’ perceptions of deserved authorship and the officially adopted definitions may be one reason why researchers sometimes end up being accused of granting undeserved authorships. However, the literature focuses mainly on other reasons. In particular, the “publish or perish” condition in modern research is seen as the main driver of deviations from good authorship practice (see [5] for a review). Together with successful funding applications, authorships of important (including highly cited) research publications are the main indicators of academic success and skill. There is therefore an incentive for researchers to obtain as many co-authorships they can, and to promote the citation and use of their research publications as much as possible. There are many ways of doing this, some of which align well with the norms of research integrity, but pressure to publish can also lead to questionable authorship practices. For instance, guest authors may be added to a research publication to promote the impact of the paper, or as “payment” for resources (e.g. tissues, equipment or man hours) provided for the study. Luiten and colleagues [21] thus point out that the literature commonly draws on a theoretical distinction between three general reasons for granting guest authorships that are not mutually exclusive: 1) Wanting to express gratitude to, or respect for, the person receiving the guest authorship, 2) wanting to promote the impact of the paper by adding a prominent name to the by-line, and 3) external pressure from a more powerful person (coerced authorship).

Given the incentive to engage in questionable authorship practices, it is not surprising that the existing literature (recently reviewed by Hosseini & Gordijn [22]) indicates that they are among the more common questionable practices–at least in the STEM sciences (Science Technology, Engineering and Mathematics) and the medical sciences. Estimates of the frequency of questionable authorship practices differ widely across studies. Studies where researchers are asked to self-report whether they have granted questionable authorships (reviewed by Marusic, Bosnjak & Jeroncic [23]) report results ranging from 1.5% to over 70%. This variance suggests that there may be interesting national and disciplinary differences in the way authorship is assigned. However, the methodological diversity of these studies makes it difficult to assess precisely how much of the variance is due to cultural differences and how much is due to different study designs.

The literature mentioned above focuses mainly on established researchers. While their perspective is clearly important, we focus on junior researchers—particularly PhD students. This is partly because they are among the most likely victims of coerced authorship, as they are at the bottom of the power hierarchy and depend on experienced researchers to build their careers.

The study reported in this paper therefore sought to contribute to a broader understanding of the problems of guest authorship by exploring and comparing the perceptions and experiences of PhD students from across the academy. The study was carried out as a questionnaire-based survey of PhD students from five European countries (Denmark, Ireland, Hungary, Portugal, and Switzerland) representing all major disciplines.

Specifically, our aim was to investigate the following research questions:

1. What proportion of the PhD students in the European countries surveyed believe they have granted guest authorships to their supervisor or other people in power?
  - Are there differences across demographic variables (age, gender, country of study) and study-specific variables (faculty, primary type of data used (e.g. qualitative, quantitative, etc.))?
2. What reasons do European PhD students give for granting guest authorships to more powerful researchers?
  - Are there differences across the demographic and study-specific variables here?
3. What are European PhD students’ views of deserved authorship?
  - Are there differences across the demographic and study-specific variables here?
  - Is there a correlation between PhD students’ perceptions of deserved authorship and their propensity to believe they have granted guest authorships to more powerful researchers?

## 2 Materials and methods

The study was based on results from a questionnaire-based survey undertaken as part of the project INTEGRITY, which aimed to map academic integrity within the European Economic Area (EEA) across three educational levels [24,25]. Prior to the survey, a qualitative interview study with *n* = 72 students including *n* = 36 PhD students was performed (details in [26]). S1 File presents the part of the questionnaire relevant to the present study. The development, translation and pilot-testing of the questionnaire are described in S2 File.

### 2.1 Ethics

The study was reviewed and approved by the Research Ethics Committee for Science and Health at the University of Copenhagen prior to the pilot tests (ref. no. 504-0043/18-5000).

All participants were over 18 years of age (see Table 2). Participation in the study was voluntary and anonymous, and the participants were not compensated. Written informed consent to participate was obtained in the first question in the questionnaire (see S1 File). Participants who did not give their consent could not continue the questionnaire.

### 2.2 Participants and recruitment

Participants included in the study were recruited in five EEA countries: Denmark, Hungary, Ireland, Portugal, and Switzerland (French-speaking part only). To ensure that we could perform the planned comparisons in a meaningful way, we aimed to recruit at least 200 participants from each country, and at least 45 from each of the following clusters (see also S3): 1) STEM and medical sciences, 2) social science, business and law, and 3) humanities and theology.

To achieve this aim, we reached out to selected PhD schools or similar institutions, asking them to help distribute the questionnaire. As explained in detail in S4, the recruitment strategy differed somewhat from country to country, partly as a result of the large variety in the numbers of PhD students and differences in the way they are institutionally organised.

In Ireland and the French-speaking part of Switzerland, a total population recruitment was carried out. All 5 universities in the French-speaking part of Switzerland, and all 9 universities in Ireland were invited to participate.

In Denmark, total population recruitments were carried out for clusters 2) and 3). For cluster 1) (the STEM and medical sciences) a complete list of PhD schools was compiled, and a random draw of schools to invite was performed.

In Portugal, a complete list of PhD programmes was compiled and sorted in accordance with the clusters mentioned above. From these lists, institutions from each faculty were randomly drawn and invited to participate.

In Hungary, five universities per cluster were randomly selected. Then, all doctoral schools in the selected universities were listed, and five doctoral schools in each university offering doctoral programmes in the given faculty were randomly selected (if there were five or fewer all were invited). All students from the selected doctoral schools were contacted.

All of the participants were recruited using standard email invitations sent out by their institution containing a link to the online survey. In some cases, reminder emails were sent after the initial invitation had been circulated. If the first round of invitations did not result in a sufficient number of participants, additional institutions were drawn based on estimates of response rates and the number of students per institution. An overview of the total number of participants invited and the resulting responses is given in Table 1.

**Table 1:** Overview of the population and sample population.

	Estimated number of students invited*	Total participants	Estimated response rate
Denmark	4,200	427	10%
Hungary	2,100	221	11%
Ireland	6,500	245	4%
Portugal	5,000	241	5%
Switzerland	3,300	202	6%
Total	21,100	1,336	6%

*Equal to the total number of students in the participating programmes (see S5).

As is evident from Table 1, the response rates were low. The consequences of this limitation are discussed in Section 4.

### 2.3 Data collection

Survey data were collected between February and December 2020. Data were collected in nine EEA countries. Thus, in addition to the five included in the study, they were collected in Lithuania, Slovenia, Germany, the Netherlands and the German-speaking part of Switzerland. However, we did not achieve sufficiently large samples (as judged against the criteria mentioned above) in these countries/areas (Lithuania: *n* = 64, Slovenia: *n* = 48, Germany: *n* = 85, the Netherlands: *n* = 171 (with *n* = 129 from the STEM and medical sciences), German speaking parts of Switzerland: *n* = 59) and they were therefore excluded. Hence the final dataset consisted of *n* = 1,336 responses from five EEA countries representing all of the major academic disciplines (S3 File contains a detailed overview of the disciplines represented).

The participants’ distribution across gender, age and faculty is shown in Table 2.

**Table 2:** Participants’ distribution across gender, age and academic field—Per country and total.

	Age (in years)			Gender distribution			Faculty					
	20th percentile	Median	80th percentile	Male	Female	Other*	STEM	Med Sci	Soc Sci	Hum	Law	Other
Denmark	27	29	34	41%	54%	5%	22%	35%	28%	10%	4%	2%
Hungary	26	29	36	38%	53%	10%	29%	22%	23%	21%	5%	1%
Ireland	26	30	42	35%	60%	6%	44%	11%	22%	20%	3%	1%
Portugal	28	34	46	36%	56%	8%	25%	12%	38%	19%	4%	2%
Switzerland	26	29	32	36%	59%	5%	27%	7%	21%	33%	9%	2%
Total	26	30	38	38%	56%	6%	29%	20%	27%	18%	5%	2%

* None of these / did not wish to answer.

Comparing the demographics of the sample with demographic information about the target populations compiled by the Organisation for Economic Co-operation and Development (OECD) [27, Indicator B7], we found that, while the age distributions in the sample roughly reflected those in the relevant populations, females were slightly overrepresented in our sample. From the OECD data we would expect roughly half of the sample to identify as female, whereas we found that 56% did so even though, unlike the OECD, we included a third gender category [27, Table B7.1]. We note, however, that this comparison can only be used as an overall indication of possible sample selection bias. Thus, as described in Section 2.2, our aim was to recruit a relatively balanced number of participants from the main faculties. Since the number of PhD students in these faculties are very disparate, with around half of all PhD students in the EU working in STEM, and less than 10% working in Law [27, Indicator B7], this could also affect the expected share of other demographic variables insofar as the gender balances and age distribution vary across faculties.

### 2.4 Materials and measures

#### 2.4.1 Demographics and study-specific factors

The questionnaire (S1 File) included questions about demographics (gender, age, country of study), and study-specific details (faculty, type of data primarily used). Descriptive details of all of the demographic and study-specific factors are given Table 1 in S5 File.

#### 2.4.2 Frequency of, and reasons for granting, guest authorship

To investigate the first research question, we asked participants to self-report whether they had allowed people “in power” to become co-authors of papers to which they had not made a significant contribution (see Table 3). (We cannot rule out that the use of the wording “in power” could lead the participants to focus overly on coercive situations between their superiors/supervisors and themselves when responding to the question. It is unclear whether memory retrieval of such coercive situations would prompt more participants to respond affirmatively, or the opposite. It may have been more neutral to refer to “in authority” instead of “in power” (we thank a reviewer for this suggestion). However, due to the actual wording of our questionnaire, we chose to keep the terminology with the present disclaimer that people in power may in many cases exercise their power based on an authority that they rightly have.) To probe the reasons for this–i.e. our second research question–we asked those who had indicated that they had granted a guest authorship to a person in power at least once to indicate their reasons for doing so (Table 3). The reasons listed as answer options were derived mainly from the initial qualitative study, but also included reasons given by students attending courses on responsible conduct of research taught by two of the authors.

**Table 3:** Survey questions and answer options probing the first two research questions.

**Research question 1: How many European PhD students believe they have granted guest authorships to their supervisor or other people in power?**	
Survey question (put to all participants)	Answer options (single choice)
During your PhD, have you allowed research group leaders, supervisors or others in power to become co-authors of papers, even though they did not make a significant contribution to them?	Yes, many times Yes, a few times Yes, once No I prefer not to answer Not applicable I don’t know
**Research question 2: What reasons do European PhD students give for granting guest authorships to more powerful researchers?**	
Survey question (put to participants who answered “yes” in some form to the question above)	Answer options (One or more options)
Which of the following best describes your reason for doing so?	The person in power told me to. I feared I would not be awarded my degree if I didn’t. Everyone else in my field does it. Friends and/or family encouraged it directly or indirectly. I believed they deserved it. I wanted to maintain a good relationship with the person. Other reasons. I prefer not to answer.

#### 2.4.3 Views of deserved authorship

The third research question was investigated using a scenario inspired by Shamoo & Resnik [28]. The scenario introduced a research project and potential co-authors, and we then asked, for each of the potential co-authors, whether it was acceptable to add them on the final paper. Given the significant variation in the types of data and materials PhD students from different faculties collect, data-specific versions of the scenario were constructed. For example, participants working primarily with quantitative data (defined in S3 File) were asked to consider the following:

“You are finalising a research paper reporting on a study that you were in charge of. The study tests a novel hypothesis using data from two different sources. An additional four people were involved in the study in various ways. For each person, please indicate whether you believe it would be acceptable to add him or her as a co-author of the paper.”

This was followed by a description of the contribution by the four potential co-authors: Dr. Doe, Dr. Jones, Dr. Santos, and Ms. Olsson. The participant was asked to respond on a five-point Likert scale ranging from “completely unacceptable” over “neutral” to “completely acceptable”. There was also an option to answer “I don’t know”.

The versions of the scenario differed as follows. The second sentence in the introduction quoted above and the descriptions of the contributions of the potential co-authors were versioned according to the type of data the participant was mainly working with. For instance, for participants working primarily with qualitative data, the second sentence in the introduction quoted above was replaced with: “The study explores a novel research topic based on qualitative data obtained from two different locations”. The version for participants working primarily with historical sources and works of art and crafts were presented with almost the same sentence, except that in these “qualitative data” was replaced with “material (sources and/or artefacts)” and “works of art”, respectively. For participants indicating that they did not work with data, the sentence read: “The project explores a novel research topic that expands on aspects of the known literature.” Full details are set out in S1 File.

The differing descriptions of the contributions of the four collaborators are shown in Table 4.

**Table 4:** Descriptions of the contributions of different collaborators in the authorship scenarios.

Contributor	Quantitative data	Qualitative data	Historical sources	Works of art	No data
**Dr. Doe**	Is your primary supervisor, he suggested the hypothesis to be tested, helped you design the study, and gave critical comments on the first draft of the publication, but did not write anything.	Same, except “hypothesis to be tested” was replaced with “research topic”.	Same as qualitative data.	Same as qualitative data.	Same as qualitative data. Except “design the study” was replaced with “frame the study”.
**Dr. Jones**	Is your secondary supervisor. You visited Dr. Jones’ research group during the study and collected some of the data you used in the study during your visit. Dr. Jones has read the manuscript and provided critical comments.	Same, except “data” was replaced with “material”.	Same as qualitative data.	Same, except “material” was replaced with “works of art”.	Same, except “collected some of the data” was replaced with “identified some of the papers”.
**Dr. Santos**	Performed part of the statistical analysis of the data. She provided some early input for the methods section, but has not read the full manuscript.	Same, except first sentence was replaced with “Coded some of the qualitative data.”	Same, except first sentence was replaced with”Helped you to compile background information from the archives.”	Same as historical sources.	Same, except first sentence was replaced with “Helped you to draw some of the figures used in the paper.”, and “method section” was replaced with “introduction”.
**Ms. Olsson**	Works as a technician at your institution. She taught you one of the key methods you used in connection with your data collection and contributed with an important adjustment of the method. She wrote part of the methods section and has read the full manuscript.	Works as a librarian at your institution. She taught you how to use a new computer programme to support your analysis and contributed an important adjustment of the method. She wrote part of the methods section and has read the full manuscript.	Same as qualitative data.	Same as qualitative data.	Same as qualitative data, except “analysis” and “methods section” were both replaced with “literature review”.

The scenario was designed with the ICMJE criteria for authorship in mind (see Introduction).

Assuming that all four collaborators in the scenario were willing to accept part of the responsibility for the study (the fourth ICMJE criterion), a strict reading of the ICMJE criteria would result in Ms. Olsson and Dr. Doe becoming co-authors. Dr. Jones and Dr. Santos, on the other hand, would not deserve to be co-authors, because they do not, on this reading, fulfil the first and third ICMJE criterion, respectively. Whether this is because they were not given the opportunity to fulfil the ICMJE definition, as the ICMJE stipulate they must, was not specified in the scenario. However, based on the qualitative study and the results from Johann and Mayer [20], we expected a substantial fraction of PhD students to have a more inclusive view of authorship, leading them to view the contribution by Dr Santos and Dr Jones as sufficient. However, we expected participants from the humanities, and possibly also from the parts of the social sciences working with qualitative data, to have a more writing-oriented view, and we anticipated that this would lead them to think it was unacceptable to grant anyone other than Ms. Olsson co-authorship [19].

Gender and titles are additional dimensions in the scenario. Dr. Doe is described using the academic title “Dr.” and the gender pronoun “he”. Dr Santos has the same academic title but is described using the gender pronoun “she”. Ms Olsson is not described using an academic title. Rather, she is a technician/librarian, and is described using the gender pronoun “she”. It is well known that there is a gender bias in research in the sense that female researchers tend to be perceived to be less competent [29,30], and there may also be a bias against people with no, or less prestigious, academic titles. It is beyond this paper to explore these biases in detail, but we note that they have been employed in combination to stack the odds against Ms Olsson and in favour of Dr. Doe.

### 2.5 Data analysis

For all outcome variables frequency distributions were run for the purpose of reporting the share of participants who ticked all response categories. In the analysis of guest authorship frequencies are reported for the entire study population (*n* = 1,336) and for a sub-sample of participants (*n* = 1,096). In the sub-sample of 1,096 participants we included those who answered either affirmatively (“yes”, at one of the three levels) or disconfirmed (“no”). Participants with other responses were excluded: “Not applicable” (187 removals), “I prefer not to answer” or “I don’t know” (35 removals), or if we could not identify their faculty belonging (18 additional removals). The first frequency distribution can be seen as an estimate of the share of all PhD participants who had granted a guest authorship. The second can be seen as an estimate of the share who had granted a guest authorship among PhD students that have already published, assuming that the participants providing the response “Not applicable” did so because they were not yet publishing their work.

To examine whether demographics and study-specific variables explained likelihood of granting a guest authorship in the sample of participants who had have already begun to publish (n = 1,096) we recoded the guest authorship response options into a binary outcome (0/1), where 1 indicates that the participant had granted a guest authorship one or more times (“yes”) and 0 indicates that the participant had not granted it (“no”), and conducted binary logistic regression. Country, faculty, type of data used, and gender identity were inserted as categorical predictors, and age as continuous predictor. We carried out unadjusted logistic regressions, in which each predictor variable was inserted one at a time, and a multivariable (adjusted) regression analysis, in which all demographics and study-specific variables were inserted conjointly. Odds ratios (OR) were reported. For all categorical variables, the baseline category has OR = 1, while categories with OR>1 and OR<1 indicate that the likelihood of the outcome (i.e. granting guest authorship) increases and decreases, respectively, compared with the baseline category. For the continuous variable, age, the OR represents the change in odds for one unit (i.e. year of age) change. The direction of the association is presented for all statistically significant variables, using predicted probabilities calculated from the *margins* command in Stata, from the adjusted regressions. Where the country variable was statistically significant we used the *atmeans* command in the presentation of predicted probabilities for this variable. This command sets all other predictor variables at their mean value for the entire sample. Following this, the country-specific predicted probabilities reflect the same average student in all countries. We used this procedure to rule out the possibility of country differences being an artefact of between-country differences on other demographic and study-specific variables caused by unequal distributions created by the disproportional sampling design and differential nonresponse errors across countries (see Section 2.2). The average PhD student for which we calculate the predicted probability of granting guest authorship in all countries is presented Table 2 in S5 File.

We also performed multivariable binary logistic regression analyses to examine whether the self-reported reasons for granting a guest authorship varied across demographic and study-specific factors. We did this only for the four reasons that were cited relatively frequently (> 50), and only included participants who had begun to publish their research (*n* = 1,096). Here, we also present the direction of statistically significant variables as predicted probabilities (using the margins command in Stata), and, if the country variable was significant, Stata’s *atmeans* command is used with the same average student characteristic, as set out Table 2 in S5 File.

In order to examine underlying patterns in the participants’ views about deserved authorship latent class analysis (LCA) [31] was carried out, with the responses to the four scenarios focusing on the contributions of collaborators being used as input variables. The six response options available in these scenarios were collapsed into four responses (Agree, Neutral, Disagree, Don’t know) before inserting them into the LCA. In the Results Section we present a latent class solution with four classes. The choice of four classes was based on a combination of statistical fit indices (where we report the AIC [32], the BIC [33], the sample-size adjusted BIC [34], entropy [35] and the Lo–Mendel–Rubin Likelihood Ratio Test [36] and conceptual considerations concerning the relevance of the latent classes given our research aims. We tested the fit of different number of classes (1 to 5 classes), and most of the statistical indices indicated that a solution with four classes fitted the data best. Thus, we found the lowest BIC and sample-size adjusted BIC [33] at four classes. The Lo–Mendel–Rubin Likelihood Ratio Test also suggested that four classes characterise the latent patterns best, as the model with five classes did not provide a significantly better fit. The AIC statistics, however, did not identify an optimal model after a model search with five classes. While the entropy value was optimal at three classes (0.84), it was still quite satisfactory (0.79) at the four-class solution. See S1 Table for detailed output of the statistical fit indices for all models (i.e. 1–5 classes).

We assigned all participants to the class to which they were most likely to belong (of the four possible classes) based on their responses to the four scenarios, and we used a multinominal logistic regression to determine which demographic and study-specific factors predicted class membership. The character of statistically significant associations between the four latent classes and the predictor variables were presented as predicted probabilities (using the margins command in Stata), and, if the country variable was significant, Stata’s *atmeans* command was applied. The average PhD student on which we calculate the predicted probability of latent class membership in all countries is presented Table 1 in S5 File.

Finally, we studied the association between latent views on deserved authorship (identified in the LCA) and having given a guest authorship using a crosstable where ORs were also reported, as was Cohen’s *w* effect size measure since it is suitable for goodness of fit statistics [37].

The following statistical software programmes were used: IBM SPSS 28.0.0, Stata/MP 17, and Mplus v 8.6. In all inferential tests we used a probability level of <0.05 to indicate a statistically significant difference.

## 3 Results

### 3.1 How many PhD students believe they have granted guest authorships to people in power?

Of the 1,336 participants, 28% (*n* = 377) indicated that they had granted a guest authorship to a person in power at least once during their PhD (Table 5). Focusing exclusively on the 1096 participants, who appeared to have begun to publish results from their research, around a third (34%) believed they had granted guest authorship to a person in power at least once. One in five (21%) had done it more than once, and 7% reported having done so many times (Table 5).

**Table 5:** Share of PhD students who believed they had granted a person in power a guest authorship.

During your PhD have you: “allowed research group leaders. supervisors or others in power to become co-authors of papers, even though they did not make a significant contribution to them.”	Shares of all participants. *n* = 1,336	Shares among participants who have published research results. *n* = 1,096*
Yes. many times	5.5%	6.7%
Yes. a few times	11.0%	13.3%
Yes. Once	11.6%	14.1%
No	55.2%	65.9%
Not applicable	13.4%	
I prefer not to answer	1.5%	
I don’t know	1.7%	
Total	100%	100%

* 222 participants answering “Not applicable”, “I prefer not to answer”, or “I don’t know” were removed along with additional 18 participants who could not be classified into one of the five main faculties.

In the unadjusted logistic regression analysis (Table 6) all demographic and study-specific factors except gender identification predicted granting guest authorship at a statistically significant level. In the adjusted analysis participant’s age was no longer statistically significant, whereas country (*p*<0.001), faculty (*p*<0.001) and type of data used (*p*<0.05) were.

**Table 6:** Results from unadjusted and adjusted logistic regression focusing on factors explaining whether PhD students had allowed someone in power to become co-authors of papers even though they had not made a significant contribution to them (once or several times during the past year) (*n* = 1,096)—Odds ratio (OR).

	Unadjusted				Adjusted model *			
	OR	CI (95%)		P-value	OR	CI (95%)		P-value
**Gender Identity** **(baseline: male)**	1			n.s.	1			n.s
Female	1.004	0.774	1.304		1.033	0.779	1.370	
Other/prefer not to say	0.966	0.545	1.712		1.238	0.657	2.333	
**Age**	0.979	0.963	0.995	<0.01	0.983	0.964	1.001	n.s
**Country (baseline: Denmark)**	1			<0.001	1			<0.001
Hungary	0.480	0.321	0.719		0.568	0.370	0.872	
Ireland	0.918	0.642	1.312		1.164	0.782	1.735	
Portugal	1.133	0.795	1.615		1.857	1.235	2.791	
Switzerland	0.692	0.457	1.047		1.164	0.732	1.851	
**Faculty** **(baseline: STEM)**	1			<0.001	1			<0.001
Medical sciences	1.320	0.937	1.859		1.378	0.946	2.010	
Social sciences	0.499	0.357	0.698		0.522	0.357	0.763	
Humanities	0.183	0.115	0.290		0.284	0.162	0.498	
Law	0.105	0.037	0.298		0.145	0.049	0.430	
**Type of data Used** **(baseline: Quantitative)**	1			<0.001	1			<0.05
Qualitative	0.414	0.292	0.589		0.802	0.527	1.220	
Historical/Works of art	0.152	0.088	0.262		0.402	0.208	0.779	
Other/No data	0.386	0.249	0.599		0.656	0.400	1.076	

Dependent variable was coded as 0 = did not allow; 1 = allowed one time or more.

* Model = Likelihood ratio χ^2^ = 154.00 (14). *p <* .001; Cox & Snell pseudo-R^2^ = 0.131; Nagelkerke pseudo-R^2^ = 0.181.

Table 7 shows patterns of effect for the three significant factors. It shows that participants from the medical sciences (49%) and STEM (42%) had higher propensity to grant guest authorships to people in power than those from the social sciences (27%), humanities (17%) and law (10%). Further, participants working predominantly with historical data or works of art had lower propensity to grant a guest authorship than participants working with qualitative and quantitative data (37%). PhD students working in Portugal had the highest probability of granting guest authorship (44%), followed by those working in Switzerland (33%), Ireland (33%), Denmark (30%), and Hungary (19%).

**Table 7:** Share of PhD students that believe they have granted at least one guest authorship to a person in power per faculty, country, and type of data used (*n* = 1,096).

Faculty	Share* (in %)
STEM	42%
Medical sciences	49%
Social sciences	28%
Humanities	17%
Law	10%
**Type of data used**	
Quantitative	37%
Qualitative	33%
Historical / works of art	20%
Other / no data	29%
**Country**	
Denmark	30%
Hungary	19%
Ireland	33%
Portugal	44%
Switzerland	33%

*Shares are calculated as predicted probabilities (using Stata’s *margins* command) based on the adjusted model in Table 6. Country shares (but not faculty and type of data used) are calculated using the *atmeans margins* command so that the shares reflect the same average student across countries.

### 3.2 Reasons for granting guest authorship

Participants who indicated that they had granted a guest authorship to a person in power at least once were asked about their reasons for doing so. Table 8 shows the results, both as shares of the participants who had allowed guest authorship and as shares among all PhD students who had published research.

**Table 8:** For participants who had granted a guest authorship to a person in power at least once: “Which of the following best describes your reason for doing so?”.

Reasons	Share of participants who had allowed guest authorship (*n* = 374)*	Shares among participants who had published research results (*n* = 1,096)
The person in power told me to	49%	17%
I wanted to maintain a good relationship with the person	48%	17%
Everyone else in my field does it	39%	13%
I believed they deserved it	22%	7%
I feared I would not be awarded my degree if I didn’t	8%	3%
Friends and/or family encouraged it directly or indirectly	1%	0%
Other reasons	18%	6%
Prefer not to answer	1%	0%

* Percentages sum to more than 100% because participants could indicate multiple reasons (except for the option “Prefer not to answer”, which was a single response option).

It can be seen that the most common reasons were: 1) the person in power telling the participant to add him or her as a co-author, 2) a wish to maintain a good relationship with the person in power, and 3) the belief that it was common practice within the field.

On average, participants indicated *M* = 1.86 (SD 0.87) reasons for granting guest authorship(s). Further analysis of the combinations of reasons showed that 14% of those who had indicated that they had granted at least one guest authorship to a person in power gave *only* the reason that the person in power had told them to (amounting to 28% of those who gave this reason). In addition, 46% of those who had been told to grant a guest authorship to a person in power also indicated that they had granted a guest authorship because they wanted to maintain a good relationship with the person receiving it. Only 9% of those who indicated that they had been told to grant a guest authorship to a person in power also indicated the reason “I thought that the person in power deserved it”.

Using logistic regression we investigated whether demographic and study-specific variables predicted reasons for granting guest authorship (for the four most common reasons reported) (see Table 9).

**Table 9:** Demographic and study-specific variables predictors of reason for granting guest authorship (*n* = 1,096).

Reason for granting guest authorship	Explanatory variables
The person in power told me to	Type of data used (*p*<0.05) Faculty (*p*<0.001) Age (*p*<0.05)
I wanted to maintain a good relationship with the person	Country (*p*<0.05) Type of data used (*p*<0.05) Faculty (*p*<0.01)
Everyone else in my field does it	Faculty (*p*<0.001)
I believed they deserved it	Gender (*p*<0.05) Type of data used (*p*<0.05)

We present the direction of the statistically significant associations for each of the four reasons in Tables 10–13.

**Table 10:** Share of PhD students (predicted probabilities) who granted a guest authorship because “The person in power told me to” (*n* = 1,096).

Type of data used	Probability of stating the reason
Quantitative	0.19
Qualitative	0.14
Historical sources/works of art	0.04
Other type or no data	0.14
Age	Probability of stating the reason
20^th^ percentile	0.19
50^th^ percentile (median)	0.17
80^th^ percentile	0.14
Faculty	Probability of stating the reason
STEM	0.17
Medical science	0.27
Social science	0.12
Humanities	0.08
Law	0.10

**Table 11:** Share of PhD students (predicted probabilities) that granted a guest authorship because “I wanted to maintain a good relationship with the person” (*n* = 1096).

Country	Probability of stating the reason
Denmark	0.16
Hungary	0.08
Ireland	0.13
Portugal	0.17
Switzerland	0.14
Type of data used	Probability of stating the reason
Quantitative	0.18
Qualitative	0.19
Historical sources/works of art	0.07
Other type or no data	0.11
Faculty	Probability of stating the reason
STEM	0.21
Medical science	0.21
Social science	0.13
Humanities	0.11
Law	0.03

Country shares (but not Type of data used) are calculated using the *atmeans margins* command, so that the shares reflect the same average student across countries.

**Table 12:** Share of PhD students (predicted probabilities) who granted a guest authorship because “Everyone else in my field does it” (*n* = 1,096).

Faculty	Probability of stating the reason
STEM	0.18
Medical science	0.20
Social science	0.09
Humanities	0.06
Law	0.05

**Table 13:** Share of PhD students (predicted probabilities) who granted a guest authorship because “I believed they deserved it” (*n* = 1,096).

Type of data used	Probability of stating the reason
Quantitative	0.08
Qualitative	0.12
Historical sources/works of art	0.03
Other type or no data	0.04
Gender identity	Probability of stating the reason
Female	0.06
Male	0.09
Other/prefer not to answer	0.16

About half of the participants who had indicated that they had granted at least one guest authorship to a person in power indicated that they had done so partly because they had been told to do so by the person in power (Table 8). From Table 10 it is clear that participants from the medical sciences (0.27) and STEM (0.17) were more likely to indicate this reason than those from other faculties (0.08–0.12).

Similarly for the wish to maintain a good relationship with the person, where once again, participants from the medical sciences (0.21) and STEM (0.21) were more likely to use this reason than those from other faculties (0.03–0.13) (Table 11).

Turning to the reason “Everyone else in my field does it”, here only faculty was a significant factor (Table 12), and again participants from the medical sciences (0.20) and STEM (0.18) were noticeably more likely to have used this reason than those from other faculties (0.05–0.09).

### 3.3 Four views about deserved authorship

The distribution of responses to the scenario concerning authorship attribution is shown in Table 14. A majority found it acceptable to grant authorship to each of the potential co-authors, except Dr Santos. The two collaborators explicitly satisfying the ICMJE criteria, Dr Doe and Ms Olsson, were also the two that the most participants found it acceptable or completely acceptable to grant co-authorship to, with 85% of participants finding it acceptable or completely acceptable to grant co-authorship to Ms Olsson, despite her gender and non-academic title (see Section 2.4.2).

**Table 14:** Participants’ beliefs about whether it would be acceptable to include each of the four collaborators as co-authors of a publication they had contributed to in varying degree (details in Section 2.4.2). Shares are reported as column percentages (*n* = 1,336).

	Dr Doe	Dr Jones	Dr Santos	Ms Olsson
Completely unacceptable	4	4	3	1
Unacceptable	16	21	26	5
Neutral	10	13	21	6
Acceptable	31	28	32	39
Completely acceptable	36	28	12	46
I don’t know	3	5	6	3

Although Dr Jones did not explicitly meet the first ICMJE criterion, 56% of the participants found it acceptable or completely acceptable to grant him or her co-authorship, and only one in four found it unacceptable or completely unacceptable. Dr Santos also did not explicitly satisfy the ICMJE criteria (because she had not read the final manuscript). A similar proportion of participants (29%) found it unacceptable to grant her a co-authorship, but here only 44% found it acceptable or completely acceptable to grant her a co-authorship.

Latent class analysis of the answers to the scenario gave the best fit for the four classes presented in Table 15 (see S1 Table for detailed statistics).

**Table 15:** Item response probabilities for the four latent classes (*n* = 1,336).

	Class 1: Lack of knowledge	Class 2: Writing-oriented, weak	Class 3: Writing-oriented, strong	Class 4: Inclusive
**Class proportion**	0.036	0.098	0.276	0.589
**DOE**				
Unacceptable	0.026	0.100	0.702	0.000
Neutral	0.000	0.577	0.078	0.036
Acceptable	0.243	0.323	0.206	0.961
Don’t know	0.732	0.000	0.015	0.002
**JONES**				
Unacceptable	0.083	0.181	0.768	0.039
Neutral	0.019	0.549	0.063	0.100
Acceptable	0.074	0.242	0.160	0.842
Don’t know	0.823	0.028	0.009	0.019
**SANTOS**				
Unacceptable	0.028	0.217	0.477	0.243
Neutral	0.000	0.503	0.159	0.191
Acceptable	0.071	0.280	0.338	0.527
Don’t know	0.902	0.000	0.025	0.039
**OLSSON**				
Unacceptable	0.018	0.006	0.117	0.051
Neutral	0.000	0.227	0.035	0.041
Acceptable	0.407	0.767	0.838	0.893
Don’t know	0.575	0.000	0.010	0.014

The first class (Class 1: Lack of knowledge) is characterised by a large proportion of “I don’t know” answers. This class is also the smallest, containing only 4% of the participants. Participants in the second class (Class 2: Writing-oriented, weak) primarily answered “neutral” to the first three possible co-authors, but strongly agreed that Ms. Olsson should be granted co-authorship. As Ms. Olsson was the collaborator who contributed most clearly to the writing, we characterise this class as weakly writing-oriented in the sense that contribution in the form of writing was considered central for attribution of co-authorship while the participants were neutral about other types of contribution. The third class (Class 3: Writing-oriented, strong), including 28% of the participants, agreed with Class 2 that Ms. Olsson should be granted co-authorship. However, in contrast with the second class, participants in the third class generally did not consider other types of contributions to be relevant for granting co-authorship. We therefore characterised this class as having a strongly writing-oriented view. The last and largest class (Class 4, Inclusive) included 59% of the participants. Those in this class were willing to grant authorship to all four collaborators, although some had reservations about Dr Santos. This class can be characterised as having a relatively inclusive view of authorship.

**Fig 1:**
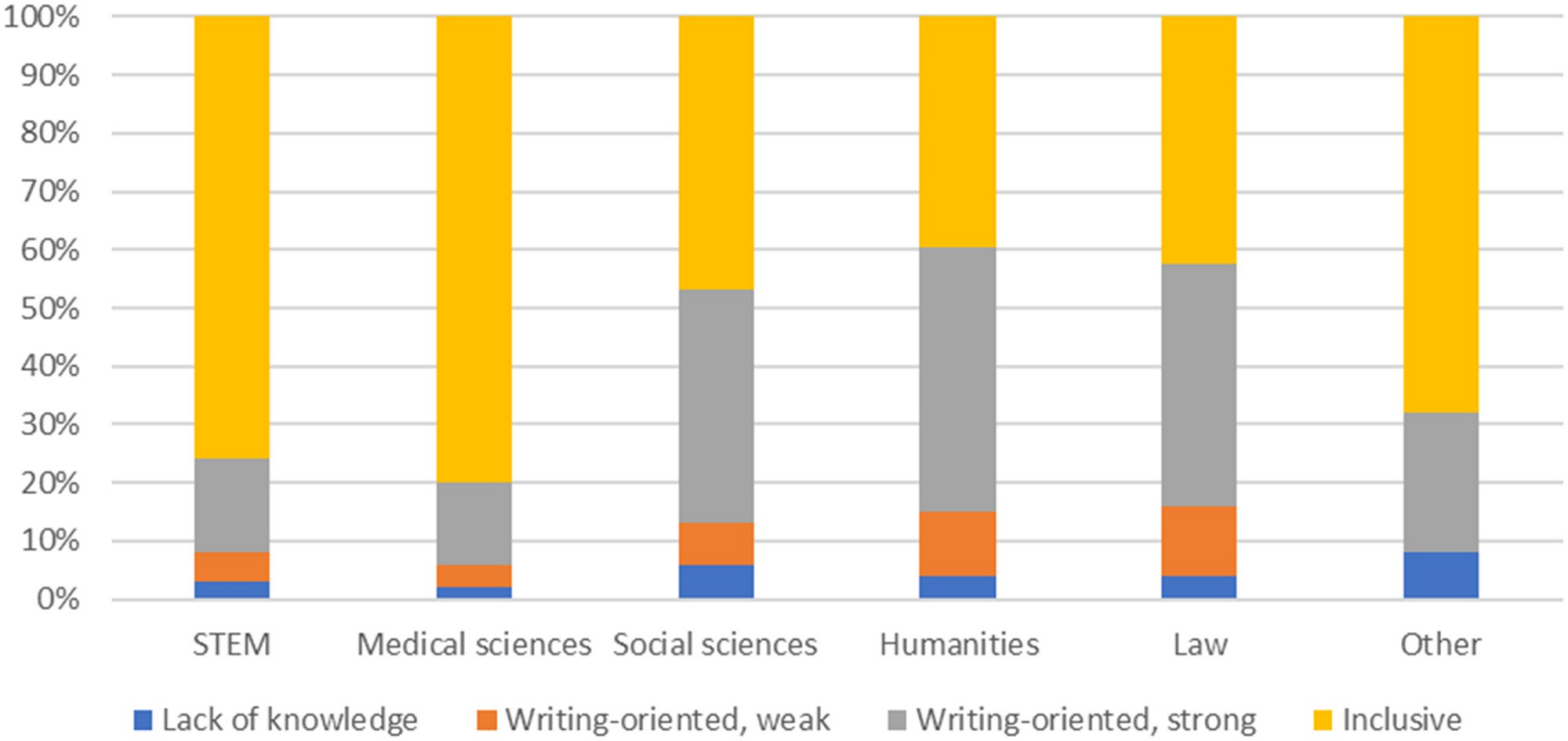
The distribution of the four views on authorship within the different faculties (shares are based on predicted probabilities).

Results from the multinomial regression analysis showed that participants from different faculties (*p* < 0.001) and countries (*p* < 0.001), as well as participants using different types of data (p < 0.001), are represented differently in the four classes. There was also a difference in views on deserved guest authorship between male and female identifying participants, and between participants who identified differently (or did not want to identify) (*p* < 0.01). As shown in Fig 1, the inclusive view dominated in the STEM (75%) and medical sciences (80%). Participants from the humanities, law and social sciences were evenly distributed between the inclusive (40–47%) and the strongly writing-oriented (40–46%) view. Also, the weakly writing-oriented view was more prevalent among participants from the humanities (11%) and law (12%) than it was among participants from STEM (5%) and medical science (6%).

**Fig 2:**
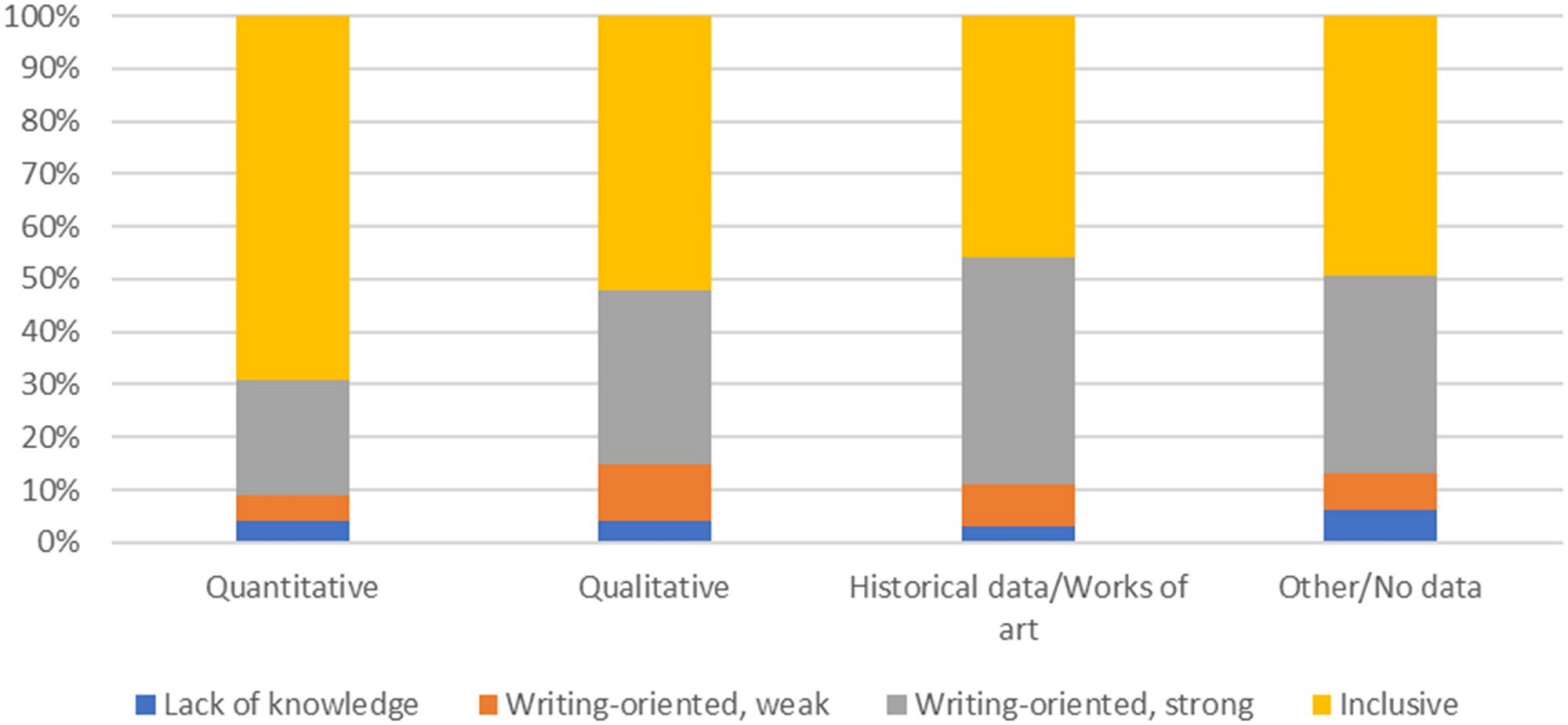
Distribution of the four views on authorship across datatypes (shares based on predicted probabilities).

The inclusive view clearly dominated among participants working with quantitative data (70%), whereas the views were more evenly distributed between the inclusive (46–52%) and strongly writing-oriented view (33–43%) among participants using other categories of data (Fig 2).

**Fig 3:**
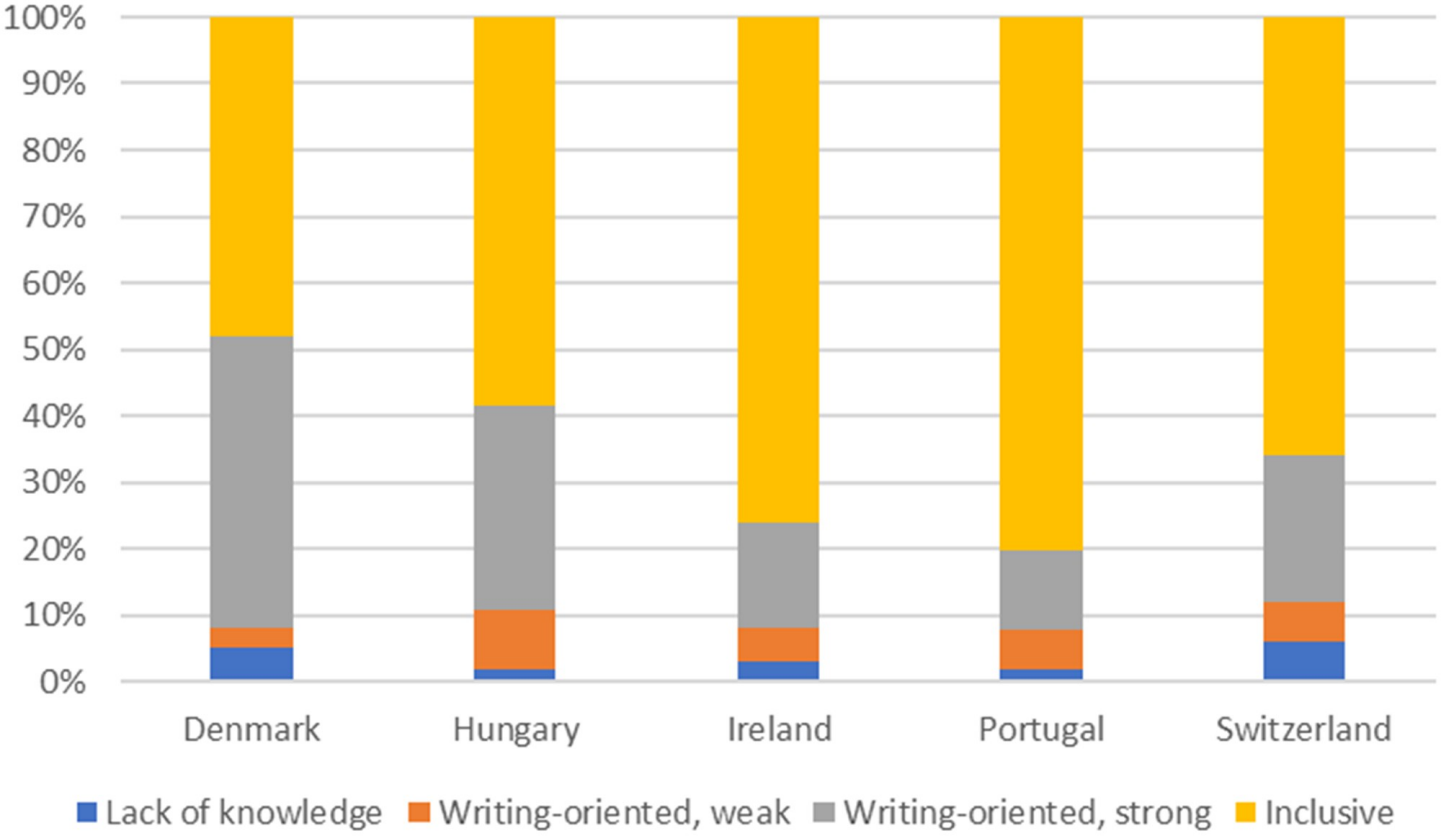
Distribution of authorship views across countries (shares calculated using the atmeans margins command to reflect the same average student across countries).

Fig 3 shows the distribution of the different views on authorship across countries. Although the inclusive view was dominant in all five countries, there were clear differences, and Denmark and Portugal stood out as extremes. In Denmark the inclusive (48%) and strongly writing-oriented (44%) views were almost evenly represented, and very few participants (3%) were weakly writing-oriented. In Portugal, a large majority (81%) held the inclusive view, and the two writing-oriented views were more equally represented (6% and 12%, respectively). The other three countries were more or the less evenly distributed between these two extremes.

**Fig 4:**
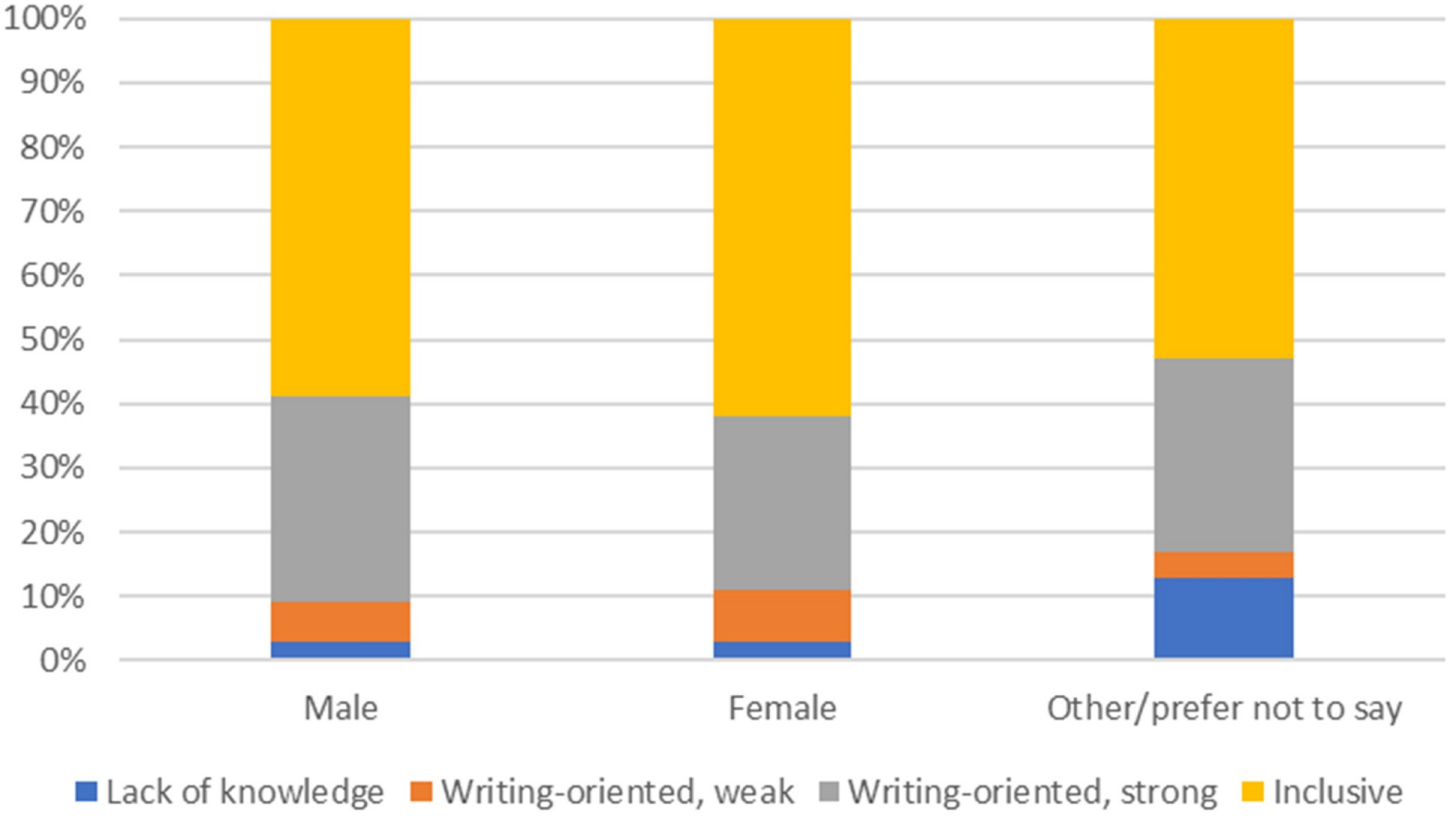
Distribution of authorship views across gender identification (shares based on predicted probabilities).

Finally, the distribution of views relative to the three gender identification options is shown in Fig 4. There was little difference between female and male identifying participants, whereas participants who identified as other or who preferred not to say were overrepresented among the participants using the “I don’t know” option.

#### 3.3.1 Relation between perception of deserved authorship and granting guest authorship

We found a statistically significant association between the likelihood that a participant would grant a guest authorship to a person in power and the view of authorship held by that participant (*p*<0.001). The association remained after we had controlled for the significant variables identified in Section 3.1 (*p*<0.001). Cohen’s *w* was 0.276, which amounts to a medium effect size [37]. As shown in Table 16, participants with an inclusive view of authorship were almost three times more likely to indicate that they have granted a guest authorship to a person in power than those holding the strongly writing-oriented view. Those holding the weakly writing-oriented view were slightly more likely than those with the strongly writing-oriented view to do so, but the difference is not statistically significant (as the 95% CI of the OR goes above and below 1). Those participants lacking knowledge were about as likely as the average participant to indicate that they have granted a guest authorship, and approximately two times more likely to do so than those holding the strongly writing-oriented view.

**Table 16:** Share granting a guest authorship to a person in power at least once across the four classes. (*n* = 1,096).

	Allowed guest authorship	OR	CI (95%)	
Writing-oriented, strong	16%	1		
Writing-oriented, weak	23%	1.5	0.82	2.82
Lack of knowledge	32%	2.5	1.10	5.56
Inclusive	44%	4.2	2.99	5.81
Total	34%			

## 4 Discussion

### 4.1 Summary of results

We set out to ascertain what share of the PhD students currently working in Denmark, Hungary, Ireland, Portugal or Switzerland believe they have granted a guest authorship to a person in power, why they did it, and how they perceive deserved authorship. We found that, if we disregard those indicating that the question does not apply to them (most likely because they had so far not published research), around one third of the participants believed they had granted at least one guest authorship to a person in power during their PhD. Participants from the STEM and medical sciences were substantially more likely to indicate that they had done so than their colleagues from other faculties. When interpreting this result it should be kept in mind that the notion of “granting” a guest authorship to a person in power covers a spectrum of actions ranging from taking an active decision alone to award a guest authorship to a person in power, to accepting a decision that was effectively made by others.

We found that almost half of those who believed they had granted guest authorship to a person in power had done so because they had been told to do so by the person in power. One in seven gave this as the only reason for granting the guest authorship. Other common reasons given included the wish to maintain a good relationship with the person in power, the perception that it is common practice in the field (“everyone else in my field does it”), and the belief that the person in power deserved it.

We identified four different views on deserved authorship: an inclusive view, a strongly writing-oriented view, a weakly writing-oriented view and the view taken by participants who indicated that they lacked knowledge about authorship attribution. In the STEM and medical sciences more than three quarters of the participants held the inclusive view, whereas less than half of participants from the social sciences, law and humanities did so. However, the writing-oriented views were much more prevalent outside the STEM and medical sciences.

Combining these findings it is noticeable that participants from the STEM and medical sciences were not only much more likely to think they had granted guest authorships to people in power but also more likely to have an inclusive view of authorship. Thus, the participants from faculties which, on average, have the most inclusive view of authorship were also the ones most likely to think that they had granted an undeserved authorship to a person in power. In other words, transgressions reported by the participants in the STEM and medical sciences were likely to be transgressions of an authorship norm that was already more inclusive than the ICMJE recommendations.

Finally, we found a strong correlation between the different views of deserved authorship and the propensity to grant a guest authorship to a person in power. Noticeably, participants holding the inclusive view were more than four times as likely to indicate that they have granted a guest authorship to a person in power as those holding the strongly writing-oriented view.

### 4.2 Comparison to previous research

As stated in the Introduction, questionable authorship practices have already been shown to be a relatively common questionable practice among experienced researchers. Our results show that granting guest authorships is also common among younger researchers.

Given that collaborative research is much more common in the STEM and medical sciences than it is in the humanities and law [38], we expected PhD students in the STEM and medical sciences to be more likely than their colleagues in the humanities and law to indicate that they have granted a guest authorship to a person in power. However, as discussed below, our results indicate that more worrying cultural differences, associated with coercive power relations, may be behind some of the differences between the faculties.

We note that, although guest authorship may be less of a problem outside the STEM and medical sciences, this does not mean that there are no problems with questionable authorship practices in these traditions. Traditions of listing only the main contributor(s) may entail failure to list people whose contribution implies that they should bear a share of the responsibility and credit for the study. In other words, more restrictive traditions may have a problem with so-called “ghost authorship”. Thus, the prevalence of the strongly writing-oriented view on authorship in the humanistic, legal and social sciences may be at least a symptom (if not a partial cause) of another problematic authorship practice namely, that PhD students working in these areas sometimes take sole credit and responsibility for research that was in reality performed in collaboration with others.

Previous studies have shown that authors working in different countries have different experiences with guest authorship [5]. For instance, there is some indication in the literature that researchers working in the Nordic countries have a lower than average tendency to grant guest authorships than their colleagues elsewhere in Europe [5]. Our study does not support this finding, at least in the case of Denmark, since our Danish results were comparable to those for Switzerland and Ireland, and PhD students working in Hungary were found to be even less likely to report that they had granted a guest authorship to a person in power. This suggests that it is not only–if it is at all–a particular culture in the Nordic countries, characterised by social-democratic welfare institutions [39], that tends to inhibit guest authorships. Portugal stands out as the country where the PhD student’s likelihood of granting a guest authorship is considerably higher. The reasons for these country differences are not clear and await further research.

Finally, type of data was a significant factor in predicting guest authorship attribution (Table 7) as well as the type of reason given (Table 9). The differences were less powerful than those across faculties, but they were retained after we controlled for faculty membership. This means that different research and data cultures also play a role in the decision to allow guest authorships. Here it is important to add that the writing-oriented view of deserved authorship was clearly less frequent among people who work with quantitative data in comparison with those who work with qualitative, historical/arts, and other/no data. To our knowledge, this is the first time that comparisons across the type of data used by a researcher have been made in connection with questionable authorship practices. Our results suggest further research into how, and why, researchers working within the same faculty, but using different methods, might have different authorship practices.

Our results on the most common reasons for granting guest authorships to people in power add to the existing literature (reviewed by Aliukonis, Poškutė & Gefenas [5]) in two ways: they incorporate data from outside the medical sciences, and they shed light on the prevalence of some of the reasons for granting guest authorships that have already been discussed in the existing literature.

As mentioned in the Introduction, the current literature tends to focus on three general reasons for granting guest authorship: 1) wanting to express gratitude and respect for the person receiving the guest authorship, 2) wanting to promote the impact of the paper by adding a prominent name to the by-line, and 3) coercive authorship [21]. Comparing to the most common reasons pointed to by our participants, we note, first, that in our study the desire to increase the impact of the paper appeared to be playing a relatively small role. We did not ask about this reason directly, but it could be one of the “other reasons” that 18% of the participants pointed to. In addition, it may be an important reason why a senior researcher may want to instruct a more junior researcher to add a given person as co-author of a paper.

Secondly, we looked at two common reasons not mentioned by Luiten et al. [21]: “Everyone else in my field does it” and “I believed the person in power deserved it”. Regarding the latter, our data do not indicate what reasons–besides making a significant contribution to a paper–the participants indicating this reason deem sufficient for acceptable attributions of co-authorship. However, we note that Johann and Mayer [20] found that roughly one in five of their participants thought that “being in a management position (without making any content related or practical contribution)” *or* being “supervisor on one of the co-authors’ doctorate” was *sufficient* for co-authorship (ibid, p. 184).

The high frequency of “everyone else in my field does it” illustrates the central role that the perceptions of peer behaviour play in questionable practices (as is well established for students’ cheating behaviour [25,40,41]). Newcomers in a field may well be unclear about what constitutes appropriate conduct, and they will tend to follow the behaviour of others when the best course of action is unclear [42]. It is possible that PhD students (most commonly those in STEM and medical sciences) become embedded in cultures where descriptive norms (what other people do) and injunctive norms (what others approve and disapprove of) [43] operate together to reinforce the practice of granting guest authorship. We believe that our data back this interpretation, since many of the participants in our study pointed out that they gave an authorship because “everyone else in my field does it” (descriptive norm), and at the same time, many had an inclusive view of authorship, rendering it a socially acceptable thing to do (injunctive norm).

“Everyone else in my field does it”, and “I believed the person in power deserved it” are also both interesting reasons because they illustrate that guest authorships are not always the result of calculated, intentional decisions made by individuals wanting to exploit the system to get a personal advantage over their peers. Rather they suggest that some researchers, especially in the STEM and medical sciences, are embedded in research cultures where guest authorships are the norm, and where not granting them means going against the custom in the field, which can be difficult and personally detrimental, especially for young researchers.

About half of those who indicated that they had granted a guest authorship to a person in power indicated that they had done it at least partly because they had been told to do so by the person in power (one in seven recorded this as the *sole* reason). Although not all of these cases necessarily involved coercion, in the sense that the person in power becomes co-author against the will of the PhD student, they are cases in which the person in power failed to convey to the PhD student the reasons why he or she thought the authorship was deserved, thus leaving the student with the impression that the decision was not a result of mutual agreement, but rather a consequence of the person in power exerting his or her authority on them. That so many PhD students indicate that they had been told to grant a guest authorship to a person in power is once again an illustration that guest authorship is a cultural problem, at least in the STEM and medical sciences.

It may be objected that the claim that there is a cultural problem with guest authorship is too strong. Thus, the findings can, at least partly, be said to be based on a comparison between messy practices and a specific abstract understanding of authorship (in this case the ICMJE definition), where discrepancies between practice and definition is always interpreted as a problem for the practice (see [9] and [44]). Against this, Patience and colleagues [9] argue that the variety in authorship norms across, and even within, faculties show that the official definitions are too rigid and lack sensitivity to “the minutiae of research”, and that we should to a large extent allow for the different research cultures and groups to be pragmatic and negotiate their own standards for awarding authorship. We agree that differences in research practice mean that it is not desirable to force one specific definition of authorship on all disciplines, and as argued further below, we find it essential that authorship criteria are continuously discussed among practicing researchers. However, our results also indicate that there are reasons to be critical of some existing traditions. To the extent that research communities should be free to decide on their own norms of authorship, it should at least include the whole community, including the junior practitioners, in the process. The results presented above, indicate that this is not the case, at least in the parts of the STEM and medical sciences, as the participants in the study experienced a discrepancy between norms and practice.

Our results on the variation across faculties in participants’ views of deserved authorship largely mirror those obtained by Johann and Mayer [20]. In both studies, an inclusive and a writing-oriented view were identified, and both studies found the inclusive view to be much more common in the STEM and medical sciences than it is in other faculties (although Johann and Mayer found a larger difference between the social sciences and humanities than we did). Both studies also identify the converse pattern for the writing-oriented view [20, p. 187]. The similarity in results across the two studies–despite differences in sample and methodology–indicates that the identified trend is robust.

Finally, we found that participants’ perceptions of deserved authorship correlate with the likelihood of granting a guest authorship to a person in power. The causal relations underlying this correlation are likely to be complex. Individual PhD students are rarely in a position where the decision about who is to be co-author on a collaborative research publication is solely up to them. It is thus unlikely that their personal beliefs about fair authorship determine whether or not they grant a guest authorship, although of course those beliefs may have some influence. There may also be an element of feedback, in the sense that the actual authorship practice in the field affects the individual researcher’s views about fair authorship assignment. In addition, perceptions of deserved authorship are likely to be influenced by the precedence and pragmatics of the field, just as the practice of authorship attribution also is (see Introduction).

Among the three main faculties, the humanities stand out as less prone to the granting of guest authorships. This propensity may well be reinforced by research practice in the humanities disciplines, where idea generation and originality are central [19]. The research practice, that is, may put pressure on researchers to appear as single agents developing new ideas or theories of their own, which would clearly limit the incentive to publish with others. This contrasts with research practices in the medical and technical sciences, where the more important focus is on appropriate methodologies and validation [19]. That focus encourages collaboration with, and support from, seniors, who usually have more experience, and a better overview, of methodological issues.

### 4.3 Limitations

Several limitations of this study should be acknowledged. One of our main aims was to compare across faculties, and we therefore intentionally adopted a disproportionate sample design. However, the disproportional design meant that our sample could not be considered representative of PhD students in the study countries. Since we have shown that problems with guest authorship are more prominent in the STEM and medical sciences, we have almost certainly underestimated the prevalence of guest authorship, as STEM students are underrepresented in our sample. Furthermore, the low response rate introduces the risk of nonresponse bias. We cannot rule out the possibility that those with a particular interest in research integrity, possibly because they have personally had negative experiences in this connection, were more willing to join the study, leading in turn to an overestimation of the problem.

Adding to this uncertainty, the questionnaire did not ask whether or not the participant had already published research. We could only indirectly infer whether participants had not published research, namely if they responded “not applicable” to the question presented in Table 3. However, it is clearly possible that some of those who answered “no” to the question whether they had granted a guest authorship did so because they had not yet published. For this reason, we may have underestimated the fraction, among participants who have published research, who have granted a guest authorship to a person in power.

Finally, we assessed the frequency of guest authorships only through self-reports from relatively inexperienced researchers. No attempt was made to triangulate the result using other methods or perspectives (e.g. an assessment of author contributions to relevant papers using official definitions like the one issued by ICMJE or more assessments of more senior co-authors).

### 4.4 Perspectives

Despite its limitations, our study raises new research avenues while also pointing to the importance of continuously discussing good authorship practice and being sensitive to differences across national and disciplinary traditions in the assignment of authorship. Our study confirms that guest authorship is a significant problem, especially in the STEM and medical sciences. It also indicates that it is, to a significant extent, a cultural problem, in the sense that some PhD students operate in work environments where guest authorships are accepted practice among senior researchers who expect and, in some cases, even demand guest authorships from the junior researchers they work with. These power relations are important to keep in mind when designing efforts to promote good authorship practice.

Institutions today are being encouraged to introduce research integrity training as a means of promoting good practice [6,7]. At least in relation to authorship practice, our results show that it is important that such training is sensitive to the cultural drivers of questionable practices. Training should, therefore, not only inform junior researchers about ideals of good authorship practice, but also provide tools for navigating the dilemmas that arise when negotiations around deserved authorship happen in groups within which there are uneven power relations.

Additionally, our results indicate that if training is used in an institution as tool for promoting good authorship practice, it is important that the training does not focus only on junior researchers. The widespread practice of awarding guest authorships appears to be systemic and should be addressed at a systemic level with initiatives aimed at broader cultural change. Our investigation does not directly identify ways to do this, but based on the literature, a few suggestions can be made. To change the culture in a research institution senior researchers must also receive research integrity training [6], and the norms of good authorship practice should be made clear to all staff. In addition, initiatives should be taken to promote and support the continued discussion of good authorship practice in the labs and corridors after the training has finished [45,46]. Clearly, these actions will in many instances require support and resources from the management level.

Furthermore, one of the likely motivations researchers in power may have for claiming guest authorships lies with the crucial role the number of authorships and citations play in funding, promotion and similar decisions. A structural move away from the use of such simple metrics and towards more qualitative decision making may remove part of the motivation for engaging in unethical authorship practice. The use of detailed authorship statements can also be seen as a step in this direction.

More radical steps could also be taken. For example, it might be suggested that better authorship practices could be promoted by introducing a clear and credible system of oversight and sanctions of norm transgressions. However, such initiatives run the danger of individualizing a systemic problem and making vulnerable researchers subject of blame rather than giving them the support they need.

## Supporting information

S1 Table Fit indices. (PDF)

S1 File Questionnaire. (PDF)

S2 File Survey development, pilot tests, and translation. (PDF)

S3 File Defining types of data and faculties. (PDF)

S4 File Details on recruitment. (PDF)

S5 File Descriptive statistics. (PDF)

S6 File Minimal dataset. (CSV)

S7 File Code book. (CSV)
