# Supplementary material for: “The person in power told me to”—European PhD students’ perspectives on guest authorship and good authorship practice
Source: PLoS One. 2023 Jan 12;18(1):e0280018. doi: 10.1371/journal.pone.0280018 (PMC9836317; doi:10.1371/journal.pone.0280018)
Supplement: S1 Table — (PDF) [file pone.0280018.s001.pdf]

# S5: Fit indices statistics from latent class analysis

The latent class analysis was performed in MPlus version 8.6

**Table 1:** Statistical fit information for latent class models with 1 to 5 classes (n=1,336)

| Number of classes | Likelihood Ratio $Chi^2$ (DF) | Loglikelihood | Entropy | AIC | BIC | Sample-Size Adjusted BIC | LMRA LRT TEST |
|---|---|---|---|---|---|---|---|
| 1 | 891.033 (242) | -5110.978 | - | 10245.955 | 0308.324 | 10270.206 | P<0.000 |
| 2 | 727.690 (230) | -4830.415 | 0.772 | 9710.829 | 9840.765 | 9761.351 | P<0.05 |
| 3 | 319.715 (217) | -4626.427 | **0.837** | 9328.854 | 9526.357 | 9405.648 | P<0.05 |
| 4 | 210.762 (204) | -4571.951 | 0.791 | 9245.902 | **9510.971** | **9348.966** | **P=0.3432** |
| 5 | 173.329 (191) | -4553.234 | 0.803 | 9234.469 | 9567.105 | 9363.805 | |
