# Supplementary material for: “The person in power told me to”—European PhD students’ perspectives on guest authorship and good authorship practice
Source: PLoS One. 2023 Jan 12;18(1):e0280018. doi: 10.1371/journal.pone.0280018 (PMC9836317; doi:10.1371/journal.pone.0280018)
Supplement: S1 File — (PDF) [file pone.0280018.s002.pdf]

S1: The questionnaire

The following contains the a part of the questionnaire developed by INTEGRITY to map how students and early career researchers understand and experience academic integrity. Questions that were not posed to PhD students are not displayed – hence the occasional jumps in variable numbers. The complete questionnaire is available here: https://doi.org/10.17894/ucph.66293057-46c5-4d02-a116-dfd597ce5a78 .

The name of each column should be self-explanatory. Please note that in the first column 'variable name' a value of 'null' indicates that this part of the questionnaire does not include a question for respondents to answer. The second column indicated which questions have treated as the same question, even though they were separate in the questionnaire.

In the third column 'introduction text and question formulation' a few questions are followed by a s_XXX number. These numbers are NOT included in the text presented to respondents. They have been included in the below version in order facilitate quicker data analysis.

If the last column is blank, all respondents have been asked the question.

In the third column, all text marked by [] brackets are not part the question. These brackets are used to explain variation across question formulation to various respondent groups in the questionnaire.

Variable name	Merged variable	Introduction text and question formulation	Answer options	Population who got the question
**Null**		[Language. This question is standard in the survey program. Only the answer options need translation.]	[English], [Local language], [Local language 2 (only if needed)]	
**Null**		This survey is about appropriate and inappropriate ways to behave as a researcher or student. Maybe you have heard about someone who behaved inappropriately, for instance by copying text from others, receiving unauthorised help, or by presenting inadequate or false results in order to make a conclusion look better. We are interested in understanding what you know and think about this because we want to improve teaching.<br>The survey will take about 20 minutes to complete.		
**s_1**		The present survey is completely anonymous. It will not be possible for anyone to trace your answers back to you. You can choose to leave the survey at any point before the survey is completed after which we will not use your responses. Once you have completed the survey you cannot withdraw your answers because they are anonymous.<br><br>About the survey<br>The survey was developed by an international team including researchers from XXX. It is part of a research project called INTEGRITY, which is funded by the EU through Horizon 2020. The results of the survey will be published in international research journals	[I understand the information provided to me and wish to participate in the survey], [I do not wish to participate in the survey]	
**Null**		Before we proceed to questions about appropriate and inappropriate practice, we would first like to ask some general questions about you		
**s_2**		What is your age?	Numerical response<br>[15-99]	
**s_3**		Has a parent or legal guardian agreed to let you participate in this survey?	[Yes], [No],<br>[I don’t know]	
**s_5**		In which country are you primarily studying?	[Denmark], [Germany], [Lithuania], [Hungary], [Ireland], [The Netherlands], [Portugal], [Slovenia], [Switzerland], [Other]	
**s_6**		What is your current level of study?	*The specific formulation is left to the translator*. General definitions: [High	

Variable name	Merged variable	Introduction text and question formulation	Answer options	Population who got the question
			school] Student in a program that is a sufficient requirement for entering one or more university educations. The program cannot itself be a university education. [BA] Student in the first cycle (see http://www.ehea.info/page-three-cycle-system) defined in the Bologna declaration, enrolled in an institution that also offers Ph.D. level education, having earned at least 60 and less than 180 ECTS points. [Master's student] Student in the second cycle defined in the Bologna declaration, having earned more than 180 ECTS points. [Research master] Student in the second cycle defined in the Bologna declaration, having earned more than 180 ECTS points, enrolled in a program that involves work on a research project that will typically be continued in a Ph.D.-project. [Ph.D. student] Student in the third cycle defined in the Bologna declaration. Students at this level are preparing a dissertation based on one or more research projects guided by one or more senior researchers, [Other]	
**s_241 & s_242**	M1	**[PHD]** Did you get your first university degree…<br><br>…in the country that you are currently studying in?	[Yes], [no]	BA and PhD students.
**s_10**		At which of the following institutions are you enrolled?	[List generated from data collection plan] + [None of the above]	PhD students

Variable name	Merged variable	Introduction text and question formulation	Answer options	Population who got the question
**s_11 & s_19**	M2	[PHD] My research is primarily within…	Q1.7b.1: [Natural sciences], [Social sciences], [Engineering], [Medical sciences], [Law], [Arts, humanities and theology], [Other]	BA and PhD students
**s_20 to s_25**		Please choose the most relevant sub-category:	If [Natural sciences] in Q1.7b.1: [Life sciences (Biology, biotech, molecular biology, agriculture, food science, sports science, etc.)], [Physics, chemistry, geoscience], [Mathematics, statistics and similar], [Computer science and similar], [Cognitive science and psychology], [Interdisciplinary], [Other]<br><br>If [Engineering] in Q1.7b.1: [Bio, environmental and food engineering], [Materials science, electrical engineering, chemical engineering, construction, transport and traffic, or similar], [Systems engineering], [Mathematics, statistics and similar], [Computer science and similar], [Interdisciplinary], [Other]<br><br>If [Humanities] in Q1.7b.1: [Language and area studies], [History and art history], [Linguistics], [Archaeology], [Literature, philosophy and history of ideas, drama, music, dance, culture and gender studies], [Anthropology ethnography, ethnology], [Media and communication science], [Pedagogy and didactics], [Psychology],	PhD students

Variable name	Merged variable	Introduction text and question formulation	Answer options	Population who got the question
			[Sociology], [Design (including architecture)], [Sports science], [Computer science and similar], [Theology], [Religious studies], [Interdisciplinary], [Other]<br><br>If [Social sciences] in Q1.7b.1: [Sociology], [Political science], [Human geography and developmental studies], [Economics], [Business], [Psychology], [Sports science], [Anthropology ethnography, ethnology], [Media and communication science], [Pedagogy and didactics], [Interdisciplinary], [Culture and gender studies], [Mathematics, statistics and similar], [Other]<br><br>If [Medical sciences]: in Q1.7b.1: [Human medicine], [Public health], [Veterinary medicine], [Medical technology], [Dentistry], [Nursing], [Interdisciplinary], [Other]<br><br>If [Other] in Q1.7b.1: [Life sciences (Biology, biotech, molecular biology, agriculture, food science, sports science, etc.)], [Physics, chemistry, geoscience], [Mathematics, statistics and similar], [Computer science and similar], [Bio, environmental and food engineering], [Materials science,	

Variable name	Merged variable	Introduction text and question formulation	Answer options	Population who got the question
			electrical engineering, chemical engineering, construction, transport and traffic or similar], [Systems engineering], [Human medicine], [Public health], [Veterinary medicine], [Medical technology], [Dentistry], [Nursing], [Language and area studies], [History and art history], [Linguistics], [Archaeology], [Literature, philosophy and history of ideas, drama, music, dance, culture and gender studies], [Anthropology ethnography, ethnology], [Media and communication science], [Pedagogy and didactics], [Psychology], [Sociology], [Design (including architecture)], [Political science], [Human geography and developmental studies], [Economics], [Psychology], [Interdisciplinary], [Theology], [Religious studies], [Other]	
**s_26, s_28 & s_29**	M3	**[PHD]** As part of your research, do you collaborate or do you plan to collaborate with others for data collection, oral presentations or some form of written work, including discussing drafts with a supervisor?	[Yes], [No]	
**s_30 & s_31**	M4	**[PHD]** There are many types of data sources. What type of data do you collect or analyse in your research? If you collect multiple types, please indicate which type you primarily use.	1. [Data suitable for statistical analysis (e.g. register data, data obtained from questionnaires, clinical trials, laboratory experiments or observations of humans, animals or nature, etc.)], 2. [Personal interviews, focus groups, observations used in sociology and anthropology or similar], 3. Historical sources (visual and textual sources,	For BA and PhD students

Variable name	Merged variable	Introduction text and question formulation	Answer options	Population who got the question
			archaeological artefacts], 4. [Works of art and craft (music, paintings, architecture, etc.)], 5. [Data of a type not listed above]<br>6. [I do not work with data]	
**NULL**		In the next part of the survey, we will focus on your experience and knowledge of how to behave in some of the difficult situations that students and Ph.D. students might encounter. You will be asked about appropriate behaviour in relation to drawing on other people's work, when working with others and when collecting, analysing and presenting data.		BA and PhD students who collaborate with others and use data
**NULL**		In the next part of the survey, we will focus on your experience and knowledge of how to behave in some of the difficult situations that students and Ph.D. students might encounter. You will be asked about appropriate behaviour in relation to drawing on other peoples' work and when working with others.		BA and PhD students who collaborate with others
**NULL**		In the next part of the survey, we will focus on your experience and knowledge of how to behave in some of the difficult situations that students and Ph.D. students might encounter. You will be asked about appropriate behaviour in relation to drawing on other peoples' work and when collecting, analysing and presenting data.		BA and PhD students who use data
**NULL**		In the next part of the survey, we will focus on your experience and knowledge of how to behave in some of the difficult situations that students and Ph.D. students might encounter. You will be asked about appropriate behaviour in relation to drawing on other peoples' work.		BA and PhD students who do not collaborate or work with data
**s_32, s_35, s_37, and s_39**	M5	To what extent do you agree with the following claim:<br>I have a good understanding of the official standards of good practice that apply to me in relation to...<br>Examples of “official standards of good practice” could be rules and regulations stating what is prohibited, but it could also include guidelines and codes of conduct describing how to behave correctly.<br><br>[2.1... citation and plagiarism	[Fully agree], [Agree], [Neutral], [Disagree], [Fully disagree], [I don’t know]	

Variabl e name	Merged variable	Introduction text and question formulation	Answer options	Population who got the question
**s_33 and s_36**	M6	[INTRODUCTION TEXT IDENTICAL TO M5]<br><br>[2.1… working with others and assigning authorship	[IDENTICAL TO M5]	For respondents who collaborate with others in their studies/research.
**s_34 and s_38**	M7	[INTRODUCTION TEXT IDENTICAL TO M5]<br><br>[2.1... collection, analysis and presentation of data	[IDENTICAL TO M5]	For respondents who work with data.
**s_41, s_44, s_46 and s_49**	M8	To what extent do you agree with the following claim:<br>In general, I know how to behave in an ethically correct manner in relation to ...<br><br>[2.2... citation and plagiarism	[Fully agree], [Agree], [Neutral], [Disagree], [Fully disagree], [I don't know]	
**s_42 and s_45**	M9	[INTRODUCTION TEXT IDENTICAL TO M8]<br><br>[2.2] … working with others and assigning authorship	[IDENTICAL TO M8]	For respondents who collaborate with others in their studies/research.
**s_43 and s_48**	M10	[INTRODUCTION TEXT IDENTICAL TO M8]<br><br>[2.2] ... collection, analysis and presentation of data	[IDENTICAL TO M8]	For respondents who work with data.
**s_50, s_231, s_234 and s_237**	M11	Over the past 12 months, have you been in a situation where you were unsure how to behave in an ethically correct manner in relation to…<br><br>[2.3.1] ... citation and plagiarism	[Yes, many times], [Yes, a few times], [Yes, once], [No], [Not applicable]	
**s_51 and s_232**	M12	[INTRODUCTION TEXT IDENTICAL TO M11]<br><br>[2.3.2] … working with others and assigning authorship	[IDENTICAL TO M11]	For respondents who collaborate with others in their studies/research.
**s_52 and s_236**	M13	[INTRODUCTION TEXT IDENTICAL TO M11]<br><br>[2.3.3] ... collection, analysis and presentation of data	[IDENTICAL TO M11]	For respondents who work with data.
**NULL**		A friend of yours wants to use a paragraph from a textbook in a paper they are currently writing. The paragraph is from page 10 in a book written by J. Brown in 1981. In the book, the paragraph reads:		For PhD students

Variable name	Merged variable	Introduction text and question formulation	Answer options	Population who got the question
		"Process Y was first discovered in 1931 by the German scientist H. Neumann, and was refined in the late 50s. Its full importance was, however, only fully understood when it was described in the seminal paper by D. Dirksen in 1971."<br><br>Your friend can incorporate the paragraph into the introduction of their own text in various ways. Some of these are presented on the next pages. For each version, please indicate whether or not you believe your friend has acted in a way that is acceptable.		
**s_53 and s_57**	M14	**[PHD]** First version: In this assignment, I will focus on a new application of process Y. As is well known, the process was first discovered in 1931 by the German scientist H. Neumann, and was refined in the late 50s. Its full importance was, however, only fully understood when it was described in the seminal paper by D. Dirksen in 1971, and the process' commercial importance was discovered. In this paper, I will continue this work by showing how process Y can be used in the production of…<br><br>The original by Brown:<br>"Process Y was first discovered in 1931 by the German scientist H. Neumann, and was refined in the late 50s. Its full importance was, however, only fully understood when it was described in the seminal paper by D. Dirksen in 1971."<br><br>Please indicate whether or not you believe your friend has acted in a way that is acceptable.	[Completely acceptable], [Acceptable], [Neutral], [Unacceptable], [Completely unacceptable], [I don't know]	
**s_54 and s_58**	M15	**[PHD]** Second version: In this assignment, I will focus on a new application of process Y. As is well known, the process was first identified in 1931 by the German scientist H. Neumann, and was later refined. Its full usefulness was, however, only fully understood when it was described in the important paper by D. Dirksen in 1971, and the process' commercial importance was discovered. In this paper, I will continue this work by showing how process Y can be used in the production of…	[IDENTICAL TO M14]	

Variabl e name	Merged variable	Introduction text and question formulation	Answer options	Population who got the question
		[TEXT SIMILAR TO M14]		
**s_55 and s_59**	M16	**[PHD]** Third version: In this assignment, I will focus on a new application of process Y. As is well known, the process was first identified in 1931 by the German scientist H. Neumann, and was later refined. Its full usefulness was, however, only fully understood when it was described in the important paper by D. Dirksen in 1971 (Brown, 1981), and the process' commercial importance was discovered. In this paper, I will continue this work by showing how process Y can be used in the production of…<br><br>[TEXT SIMILAR TO M14]	[IDENTICAL TO M14]	
**s_56 and s_60**	M17	**[PHD]** Fourth version: In this assignment, I will focus on a new application of process Y. According to Brown (1981:10), the process was first discovered in 1931, although its usefulness and commercial importance were only understood in the early 1970s. In this paper, I will continue this work by showing how process Y can be used in the production of…<br><br>[TEXT SIMILAR TO M14]	[IDENTICAL TO M14]	
s_61 to s_64		Please indicate whether you believe the following actions go against the official rules and regulations that apply to you in relation to plagiarism.<br>• [Q2.6.1] Copying an entire page stating a central point from an external source into your own text without quotation marks but including a reference. s_61<br>• [Q2.6.2] Copying one short paragraph stating a central point from an external source into your own text without quotation marks but including a reference. s_62<br>• [Q2.6.3] Changing 10% of the words in a short paragraph stating a central point from an external source and using it in your own text with a reference. s_63<br>• [Q2.6.4] Copying a central point formulated in half a sentence from an external source without marking it with quotation marks but including a reference. s_64	[Yes, it is a serious violation], [Yes, but it is not a serious violation], [No, it is not against the rules], [The rules are unclear], [It depends on the situation], [I don't know]	

Variable name	Merged variable	Introduction text and question formulation	Answer options	Population who got the question
**s_65, s_69, s_73, and s_77**	M18	**[PHD – quantitative data]** You are finalising a research paper reporting on a study that you were in charge of. The study tests a novel hypothesis using data from two different sources. An additional four people were involved in the study in various ways. For each person, please indicate whether you believe it would be acceptable to add him or her as a co-author of the paper.<br><br>[Q2.10c.1] Dr. Doe: Is your primary supervisor, he suggested the hypothesis to be tested, helped you design the study, and gave critical comments on the first draft of the publication, but did not write anything.<br><br>**[PHD – qualitative data]** You are finalising a research publication reporting on a study that you were in charge of. The study explores a novel research topic based on qualitative data obtained from two different locations. An additional four people were involved in the study in various ways. For each person, please indicate whether you believe it would be acceptable to add him or her as a co-author of the publication.<br><br>• [Q2.10c.1] Dr. Doe: Is your primary supervisor, he suggested the research topic, helped you design the study, and gave critical comments on the first draft of the publication, but did not write anything.<br><br>**[PHD – historical sources]** You are finalising a research publication reporting on a study that you were in charge of. The study explores a novel research topic based on material (sources and/or artifacts) obtained from two different locations. An additional four people were involved in the study in various ways. For each person, please indicate whether you believe it would be acceptable to add him or her as a co-author of the publication.<br><br>• [Q2.10c.1] Dr. Doe: Is your primary supervisor, he suggested the research topic, helped you design the study and gave critical comments on the first draft of the publication, but did not write anything.	[Completely acceptable], [Acceptable], [Neutral], [Unacceptable], [Completely unacceptable], [I don’t know]	For PhD students

Variable name	Merged variable	Introduction text and question formulation	Answer options	Population who got the question
		**[PHD – works of art as data]** You are finalising a research publication reporting on a study that you were in charge of. The study explores a novel research topic based on an analysis of several works of art obtained from two different locations. An additional four people were involved in the study in various ways. For each person, please indicate whether you believe it would be acceptable to add him or her as a co-author of the publication.<br>• [Q2.10c.1] Dr. Doe: Is your primary supervisor, he suggested the research topic, helped you design the study and gave critical comments on the first draft of the publication, but did not write anything.<br><br>**[PHD – no data]** You are finalising a research publication reporting on a project you were in charge of. The project explores a novel research topic that expands on aspects of the known literature. An additional four people were involved in the project in various ways. For each person, please indicate whether you believe it would be acceptable to add the person as a co-author of the publication.<br><br>• [Q2.10cε.1] Dr. Doe: Is your primary supervisor, he suggested the research topic, helped you to frame the project and gave critical comments on the first draft of the publication, but did not write anything.		
**s_66, s_70, s_74, and s_78**	M19	[INTRODUCTION TEXT SIMILAR TO M18 FOR ALL VARIATIONS]<br><br>**[PHD – quantitative data]** Dr. Jones: Is your secondary supervisor. You visited Dr. Jones' research group during the study and collected some of the data you used in the study during your visit. Dr. Jones has read the manuscript and provided critical comments.<br><br>**[PHD – qualitative data]** Dr. Jones: Is your secondary supervisor. You visited Dr. Jones' research group during the study and collected some of the material you used in the study during your visit. Dr. Jones has read the manuscript and provided critical comments.	[IDENTICAL TO M18]	For PhD students

Variabl e name	Merged variable	Introduction text and question formulation	Answer options	Population who got the question
		**[PHD – historical sources]** Dr. Jones: Is your secondary supervisor. You visited Dr. Jones' research group during the study and collected some of the material you used in the study during your visit. Dr. Jones has read the manuscript and provided critical comments.<br>**[PHD – works of art as data]** Dr. Jones: Is your secondary supervisor. You visited Dr. Jones' research group during the study and collected some of the works of art you used in the study during your visit. Dr. Jones has read the manuscript and provided critical comments.<br><br>**[PHD – no data]** Dr. Jones: Is your secondary supervisor. You visited Dr. Jones' research group during the project and identified some of the papers you used in the project during your visit. Dr. Jones has read the manuscript and provided critical comments.		
**s_67, s_71, s_75 and s_79**	M20	[INTRODUCTION TEXT SIMILAR TO M18 FOR ALL VARIATIONS]<br><br>**[PHD – quantitative data]** Dr. Santos: Performed part of the statistical analysis of the data. She provided some early input for the methods section, but has not read the full manuscript.<br><br>**[PHD – qualitative data]** Dr. Santos: Coded some of the qualitative data. She provided some early input for the methods section, but has not read the full manuscript.<br><br>**[PHD – historical sources]** Dr. Santos: Helped you to compile background information from the archives. She provided some early input for the methods section, but has not read the full manuscript.<br><br>**[PHD – works of art as data]** Dr. Santos: Helped you to compile background information from the archives. She provided some early input for the methods section, but has not read the full manuscript.	[IDENTICAL TO M18]	For PhD students

Variable name	Merged variable	Introduction text and question formulation	Answer options	Population who got the question
		**[PHD – no data]** Dr. Santos: Helped you to draw some of the figures used in the paper. She provided some early input for the introduction, but has not read the full manuscript.		
**s_68, s_72, s_76, and s_80**	M21	[INTRODUCTION TEXT SIMILAR TO M18 FOR ALL VARIATIONS]<br><br>**[PHD – quantitative data]** Ms. Olsson: Works as a technician at your institution. She taught you one of the key methods you used in connection with your data collection and contributed with an important adjustment of the method. She wrote part of the methods section and has read the full manuscript.<br>**[PHD – qualitative data]** Ms. Olsson: Works as a librarian at your institution. She taught you how to use a new computer program to support your analysis and contributed with an important adjustment to your method. She wrote part of the methods section and has read the full manuscript.<br><br>**[PHD – historical sources]** Ms. Olsson: Works as a librarian at your institution. She taught you how to use a new computer program to support your analysis and contributed with an important adjustment to your method. She wrote part of the methods section and has read the full manuscript.<br><br>**[PHD – works of art as data]** Ms. Olsson: Works as a librarian at your institution. She taught you how to use a new computer program to support your analysis and contributed with an important adjustment to your method. She wrote part of the methods section and has read the full manuscript.<br><br>**[PHD – no data]** Ms. Olsson: Works as a librarian at your institution. She taught you how to use a new computer program to support your analysis and contributed with an important adjustment to the literature review. She wrote part of the literature review and has read the full manuscript.	[IDENTICAL TO M18]	For PhD students
**s_89, s_90 and s_91**		Please indicate whether you believe the following actions go against the official rules and regulations that apply to you in relation to collaboration and authorship.	[Yes, it is a serious violation], [Yes, but it is not a serious violation], [No, it is not against the rules], [The rules are	For PhD students

Variabl e name	Merged variable	Introduction text and question formulation	Answer options	Population who got the question
		• [Q2.11c.1] Adding someone who has not written anything as an author of a research publication. s_89<br>• [Q2.11c.2] Adding a supervisor who only provided critical comments on the manuscript as a co-author of the research publication. s_90<br>• [Q2.11c.3] Removing a co-author who made a genuine contribution to the writing of a manuscript because the person does not want to be a co-author. s_91	unclear], [It depends on the situation], [I don't know]	
s_92, s_98, s_101, s_104, s_107, and s_110	M22	Please indicate whether you believe the following actions go against the rules and regulations that apply to you in relation to data collection, analysis and presentation.<br><br>**[QUANTITATIVE DATA]** Not mentioning in an assignment that you removed a number of deviating data points from a data set when the cause of the deviation was unknown.<br>**[PhD formulation]** Not mentioning in a research publication that you removed some deviating data points from a data set when the cause of the deviation was unknown.<br><br>**[QUALITATIVE DATA]** Not mentioning in an assignment that you discarded one or more interviews that did not fit well with the rest of your interviews when the cause of the deviation was unknown.<br>**[PhD formulation]** Not mentioning in a research publication that you discarded one or more interviews that did not fit well with the rest of your interviews when the cause of the deviation was unknown.<br><br>**[ARTIFACTS/SOURCE DATA]** Not mentioning in an assignment that you discarded some of your material that did not fit well with the rest of your material when the cause of the deviation was unknown.<br>**[PhD formulation]** Not mentioning in a research publication that you discarded some of your material that did not fit well with the rest of your material when the cause of the deviation was unknown.	[Yes, it is a serious violation], [Yes, but it is not a serious violation], [No, it is not against the rules], [The rules are unclear], [It depends on the situation], [I don't know]	For respondents who work with data.

Variable name	Merged variable	Introduction text and question formulation	Answer options	Population who got the question
s_93, s_99, s_102, s_105, s_108, and s_111	M23	**[INTRODUCTION SAME AS M22]**<br><br>**[FOR PHD STUDENTS ASSIGNMENT IS REPLACED WITH RESEARCH PUBLICATION]**<br><br>**[QUANTITATIVE DATA]** Not mentioning in an assignment that you removed a number of deviating data points from a data set when the cause of the deviation was known.<br>**[PhD formulation]** Not mentioning in a research publication that you removed some deviating data points from a data set when the cause of the deviation was known.<br><br>**[QUALITATIVE DATA]** Not mentioning in an assignment that you discarded one or more interviews that did not fit well with the rest of your interviews when the cause of the deviation was known.<br>**[PhD formulation]** Not mentioning in a research publication that you discarded one or more interviews that did not fit well with the rest of your interviews when the cause of the deviation was known.<br><br>**[ARTIFACTS/SOURCE DATA]** Not mentioning in an assignment that you discarded some of your material that did not fit well with the rest of your material when the cause of the deviation was known.<br>**[PhD formulation]** Not mentioning in a research publication that you discarded some of your material that did not fit well with the rest of your material when the cause of the deviation was known.	[SAME AS M22]	For respondents who work with data.
s_94, s_100, s_103, s_106, s_109, and s_112	M24	**[INTRODUCTION SAME AS M22]**<br><br>**[FOR PHD STUDENTS ASSIGNMENT IS REPLACED WITH RESEARCH PUBLICATION]**<br><br>**[QUANTITATIVE DATA]** Not mentioning in an assignment that you replaced a number of outliers in a data set with data points obtained through estimates based on the remaining data points.	[SAME AS M22]	For respondents who work with data.

Variable name	Merged variable	Introduction text and question formulation	Answer options	Population who got the question
		**[PhD formulation]** Not mentioning in a research publication that you replaced some outliers in a data set with data points obtained through estimates based on the remaining data points.<br><br>**[QUALITATIVE DATA]** Not mentioning in an assignment that you made up a number of direct quotes from an interview based on your general impression of the interviewee's viewpoints.<br>**[PhD formulation]** Not mentioning in a research publication that you made up a number of direct quotes from an interview based on your general impression of the interviewee's viewpoints.<br><br>**[ARTIFACTS/SOURCE DATA]** Not mentioning in an assignment that you made up a number of quotes based on your general impression of the viewpoints expressed in a source.<br>**[PhD formulation]** Not mentioning in a research publication that you made up a number of quotes based on your general impression of the viewpoints expressed in a source.		
**NULL**		In the following section, we will ask you some questions about how and where you obtained your knowledge about good practice.		
s_113		Have you taken courses on rules and/or ethically correct behaviour in relation to the themes introduced above during your current or previous studies? (Multiple answers possible)	[Yes, one or more dedicated courses], [Yes, one or more lectures], [Yes, one or more dedicated e-learning sessions], [No]	
s_114		Approximately how many working days have you spent on such courses in total?	[Less than 1 working day], [1 full working day], [1-3 full working days], [3-5 full working days], [1-2 working weeks], [More than 2 working weeks], [I do not recall]	
s_116		Have you learned about rules and/or ethically correct behaviour in relation to the themes introduced above through any other method?<br>(Multiple answers possible)	[Yes, through supervisors/teachers in other courses that commented on my written work or assignments], [Yes, through courses not dedicated exclusively to such issues], [Yes,	For BA and PhD students

Variabl e name	Merged variable	Introduction text and question formulation	Answer options	Population who got the question
			through discussions with fellow students], [Yes, through discussions with senior staff outside regular courses], [Yes, through self-study], [Yes, by following the procedures that are common in my field of study], [Yes, through discussions with friends and family outside my institution], [Yes, other], [No], [I don’t know]	
s_118		Below are a number of topics related to appropriate and inappropriate ways of behaving as a researcher. Please indicate the **three topics** you think are the most important when such themes are taught in your field of research.	[Drawing on other peoples' work (including plagiarism)], [Working with others], [Collecting, analysing and presenting data], [privacy and confidentiality], [The social responsibility of science and scientists], [Pressure from supervisors or others in power], [Conflicts of loyalty], [Guidelines on scientific/research integrity], [Responsibility for human subjects/informants], [Responsible data storage and management], [Conflicts of interest], [How to deal with violations and allegations of misconduct], [Research culture (e.g. elements in the research environment that promote or hinder responsible conduct)], [Strategies for dealing with dilemmas in research], [Ethical issues related to peer reviewing], [Protection of animal subjects], [Other topics], [I don’t think teaching about such themes is relevant], [I don't know].	For PhD students

Variabl e name	Merged variable	Introduction text and question formulation	Answer options	Population who got the question
NULL		It is one thing to know the right thing to do in a given situation, but another to actually do it. You have probably heard rumours about researchers and/or students who plagiarised, manipulated data or deviated from ideal practice in other ways. We are interested in knowing how common you think such deviations are among your peers and whether you yourself have deviated from best practice. We remind you that this survey is entirely anonymous; no one will be able to identify your specific answers to these questions.		
s_122 s_127, s_133, s_136, s_143, s_148, s_151	M28	**To what extent do you agree with the following statements?**<br>**It is common for my classmates to...**<br><br>**[FOR PHD STUDENTS SUBSTITUTE 'TEXTS' WITH 'RESEARCH PAPERS']**<br><br>[Q4.1bα.4] ... copy shorter passages from other sources into their own texts without marking them as quotes.	[SAME AS M25]	
s_123 s_128, s_134, s_137	M29	[INTRODUTION TEXT SAME AS M25]<br><br>[Q4.1bα.5] … add students as co-authors of group assignments, even though they did not contribute.	[SAME AS M25]	
s_139, s_149, s_152	M31	To what extent do you agree with the following statements?<br>It is common for researchers in my field to...<br>[Q4.1cα.1] ... allow research group leaders, supervisors or others in power to become co-authors of papers, even though they did not make a significant contribution to them.	[Fully agree], [Agree], [Neutral], [Disagree], [Fully disagree], [I don't know]	For Phd students
s_144, s_150, s_153	M32	[INTRODUCTION TEXT SAME AS M31]<br><br>[Q4.1cα.6] ... cite sources that are not strictly relevant in order to please a reviewer or in the hope that the author of the source might return the favour.	[SAME AS M31]	For PhD students
s_154, s_160, s_168, s_189, s_198	M33	[FOR HIGH SCHOOL STUDENTS 'university' IS REPLACED WITH 'high-school']<br>During your university education, have you...<br>(We remind you that this survey is entirely anonymous; no one will be able to identify your specific answers to these questions)	[Yes, many times], [Yes, a few times], [Yes, once], [No], [I prefer not to answer], [Not applicable], [I don't know]	All respondents who work with data

Variable name	Merged variable	Introduction text and question formulation	Answer options	Population who got the question
		**[QUANTITATIVE DATA]** ... deleted deviating data points based on a gut feeling that they were inaccurate.<br><br>**[NON-QUANTITATIVE DATA]** ... ignored parts of your material that did not fit well with the rest of the material based only on a gut feeling that the deviating material was unreliable.		
s_161, s_169, s_190, s_199	M38	[INTRODUCTION TEXT SAME AS M33]<br><br>**[FOR PHD STUDENTS SUBSTITUTE 'TEXTS' WITH 'RESEARCH PAPERS']**<br><br>**[QUANTITATIVE DATA]** ... performed a misleading or dubious statistical analysis in order to achieve results that a teacher would accept.<br><br>**[NON-QUANTITATIVE DATA]** ... performed misleading or dubious interpretation of sources, artifacts or qualitative data in order to achieve results a teacher would accept.	[SAME AS M33]	All respondents who work with data
s_162, s_170, s_191, s_200	M39	[INTRODUCTION TEXT SAME AS M33]<br><br>[Q4.2α.3] **... kept inadequate records of parts of their work that should be documented** (e.g. laboratory records, descriptions of data sampling procedures, key words used in a literature study, types of statistical tests performed, etc.).<br>**[QUANTITATIVE DATA]**<br><br>**[NON-QUANTITATIVE DATA]** ... kept inadequate records of parts of your work that should be documented (e.g. descriptions of data sampling procedures, key words used in a literature study, procedure for qualitative coding etc.).	[SAME AS M33]	All respondents who work with data

Variable name	Merged variable	Introduction text and question formulation	Answer options	Population who got the question
s_156, s_163, s_171, s_183, s_192, s_201, s_204	M40	[INTRODUCTION TEXT SAME AS M33]<br><br>[Q4.2bα.4] ... copied shorter passages from other sources into your own text without marking them as quotes.	[SAME AS M33]	
s_188, s_197, s_203	M45	During your Ph.D., have you...<br>(We remind you that this survey is entirely anonymous; no one will be able to identify your specific answers to these questions)<br><br>[Q4.2cα.1] ... allowed research group leaders, supervisors or others in power to become co-authors of papers, even though they did not make a significant contribution to them.	[Yes, many times], [Yes, a few times], [Yes, once], [No], [I prefer not to answer], [Not applicable], [I don't know]	PhD students
s_196, s_202, s_208	M46	[INTRODUCTION TEXT SAME AS M45]<br><br>[Q4.2cα.6] ... cited sources that were not strictly relevant in order to please a reviewer or in the hope that the author of the source might return the favour.	[SAME AS M45]	PhD students
**NULL**		The last questions are about *why* people deviate from best practice. There may of course be many reasons, but we are interested in knowing which ones you think are the most important.		
s_209, s_219	M47	To what extent do you agree with the following statements?<br>If students at my institution deviate from what is ethically acceptable (for instance by plagiarising or putting their name on work they did not contribute to), they do it because...<br>[For PhD students, second paragraph reads: "If Ph.D. students at my institution deviate from best practice, e.g. plagiarise, offer undeserved authorships, handle and analyse data poorly etc., they do it because…"]<br><br>• [Q5.1ab.1] ... they are lazy.	[Fully agree], [Agree], [Neutral], [Disagree], [Fully disagree], [I don't know], [Not applicable]	

Variable name	Merged variable	Introduction text and question formulation	Answer options	Population who got the question
s_210, s_220	M48	[INTRODUCTION TEXT SIMILAR TO M47]<br><br>[Q5.1ab.2] ... their teacher or other people in power tell them to	[SAME AS M47]	
s_211, s_221	M49	[INTRODUCTION TEXT SIMILAR TO M47]<br><br>[Q5.1ab.3] ... they fear they will not be awarded their degree if they don't.	[SAME AS M47]	
s_212, s_222	M50	[INTRODUCTION TEXT SIMILAR TO M47]<br><br>[Q5.1ab.4] ... they think it is very unlikely that they will get caught.	[SAME AS M47]	
s_213, s_223	M51	[INTRODUCTION TEXT SIMILAR TO M47]<br><br>[Q5.1ab.5] ... they think everyone else does it	[SAME AS M47]	
s_214, s_224	M52	[INTRODUCTION TEXT SIMILAR TO M47]<br><br>[Q5.1ab.6] ... their friends and/or family encourage it directly or indirectly.	[SAME AS M47]	
s_215, s_225	M53	[INTRODUCTION TEXT SIMILAR TO M47]<br><br>[Q5.1ab.7] ... they want to be the best.	[SAME AS M47]	
s_216, s_226	M54	[INTRODUCTION TEXT SIMILAR TO M47]<br><br>[Q5.1ab.8] ... they have not been properly informed what the ethically correct behaviour is.	[SAME AS M47]	
s_218, s_228	M55	[INTRODUCTION TEXT SIMILAR TO M47]<br><br>[Q5.1ab.10] … other reasons. s_218	[SAME AS M47]	
s_229		You stated that on at least one occasion, you allowed a research group leader, supervisor or another person in power to become co-author of one of your research publications, even though they did not make a significant	[The person in power told me to.],<br>[I feared I would not be awarded my degree if I didn't.],	For PhD students who have at least once allowed a person in power to be

Variable name	Merged variable	Introduction text and question formulation	Answer options	Population who got the question
		contribution to the research publication. Which of the following best describes your reason for doing so?<br>(Multiple answers possible)	[Everyone else in my field does it.], [Friends and/or family encouraged it directly or indirectly.],<br>[I believed they deserved it],<br>[I wanted to maintain a good relationship with the person], [Other reasons], [I prefer not to answer]	co-author on a publication despite this person not contributing significantly to said publication.
**6.0 [1.4]**		Before you submit your data we would like to ask you one final question: Which gender do you primarily identify with?	[Male], [Female], [None of the above], [I prefer not to answer]	
		The survey is now finished. Thank you very much for your participation! If you are interested in the results of this survey, you will be able to find them at http://h2020integrity.eu/ as soon as they are available.		
**PX**		Unfortunately, you are outside the target group of this survey. Thank you for your time. If you are interested in the results of this survey, you will be able to find them at http://h2020integrity.eu/ as soon as they are available.		
