# Supplementary material for: “The person in power told me to”—European PhD students’ perspectives on guest authorship and good authorship practice
Source: PLoS One. 2023 Jan 12;18(1):e0280018. doi: 10.1371/journal.pone.0280018 (PMC9836317; doi:10.1371/journal.pone.0280018)
Supplement: S2 File — (PDF) [file pone.0280018.s003.pdf]

S2: Survey development, pilot tests, and ethics

This appendix is based partly on the corresponding appendix in [1] which reports on a different subset of the larger dataset on which the present study is also based.

As mentioned in the main text, the survey was run within the INTEGRITY project, and thus it was framed in accordance with the general empirical aims of that project:

1. **identify, map and evaluate** the existing understanding of students and early-career researchers in terms of academic and research integrity and key concepts in relation to questionable research practice and research misconduct, including fabrication, falsification, and plagiarism via a new survey tool developed for this purpose;
2. **map and categorise** the grey area issues relating to good scientific practice that students and early-career researchers are most likely to face in their daily practice, and the extent to which these vary across cultural and disciplinary backgrounds.

These general aims were translated into 15 research questions that guided the design of the study. Note that the INTEGRITY project as a whole covers three educational levels: upper secondary school, undergraduate and PhD, but this paper reports only on selected research questions targeting the PhD sub-population.

The survey was not intended to answer all the research questions generated, but, broadly speaking, the combination of the survey and interview studies should answer all of them.

The qualitative leg of the study is described in detail in [2]. In the interview study, we identified, among other things, the reasons listed as answer options listed in Table 3 of the main text to the question about why PhD students award guest authorships to people in power.

1 Survey development and pilot testing

Drawing on the interview study, existing literature and the above-mentioned research aims, a pilot survey was developed by the team at the University of Copenhagen.

The pilot survey (in English) was circulated to all participating countries to ensure that all of the questions were relevant and meaningful in all of the relevant didactical contexts. Following revisions, the survey was set up online and pilot-tested.

Two types of pilot test were performed. First, a small qualitative test was run. This involved a few local PhD students, and a handful of Danish undergraduate and upper secondary school students, all of whom were asked to think aloud while answering the pilot survey, followed by a debriefing on what they found difficult or unclear. Second, the pilot survey was translated into Dutch, Lithuanian and Portuguese, and then distributed to 550 students recruited using convenience sampling. The pilot data were analysed to check whether any of the questions were too difficult, or too similar to other questions, to allow for meaningful analysis. We also sought to reduce the completion time to improve the response rate.

Following the pilot tests, a number of questions were cut from the questionnaire. Others were merged, and the language was generally simplified.

2 Translation of the final survey

When the final English version of the survey was ready it was translated into the main languages in the nine countries initially participating in the study. To ensure comparability across languages, we used a translation-back-translation approach: for each language, the survey was first translated into the intended language, then back-translated into English by an independent translator without access to the original. The first author of this paper then compared the back-translation with the original version and discussed any discrepancies with the relevant partners in order to identify any necessary adjustments to the translated versions.

Once all versions were ready, the online survey was set up in a way that allowed participants in any country to choose between an English version and a version translated into the language of their country of study. Native-speaking project members thoroughly and systematically tested each version of the electronic survey, using a list of predefined personas and looking for language and technical errors, before it was sent to participants.

References:

[1] Johansen MW, Goddiksen MP, Centa M. Clavien C, Gefenas E, Globokar R, et al. Lack of ethics or lack of knowledge? European upper secondary students' doubts and misconceptions about integrity issues. Int J Educ Integr 2020;**18(**20). Doi: https://doi.org/10.1007/s40979-022-00113-0.

[2] Goddiksen M, Quinn U, Kovács N, Lund TB, Sandøe P, Varga O, et al. Good friend or good student? An interview study of perceived conflicts between personal and academic integrity among students in three European countries. Accountability in Research 2021;28:4: 247-264. DOI: 10.1080/08989621.2020.1826319
