# Supplementary material for: “The person in power told me to”—European PhD students’ perspectives on guest authorship and good authorship practice
Source: PLoS One. 2023 Jan 12;18(1):e0280018. doi: 10.1371/journal.pone.0280018 (PMC9836317; doi:10.1371/journal.pone.0280018)
Supplement: S3 File — (PDF) [file pone.0280018.s004.pdf]

S3: Types of data and faculties

1 Types of data types

In the questionnaire (S1), some questions were tailored to the types of data with which the relevant participants were primarily working. Differences across types of data were also investigated in the data analysis. The questionnaire distinguished four types of data, and it included two further responses – one indicating an alternative data source and the other indicating that the participant was not working with data in their PhD.

Table 1: There are many types of data sources that can be used in research. What type of data do you collect or analyse in your research? If you collect multiple types, please indicate which type you primarily use.

Datatype	n	Percent
1 Data suitable for statistical analysis (e.g. register data, data obtained from questionnaires, clinical trials, laboratory experiments or observations of humans, animals or nature, etc.)	747	55.9
2 Personal interviews, focus groups, observations used in sociology and anthropology or similar	262	19.6
3 Historical sources (visual and textual sources, archaeological artefacts)	148	11.1
4 Works of art and craft (music, paintings, architecture, etc.)	27	2.0
5 Data of a type not listed above	95	7.1
6 I do not work with data	57	4.3
Total	1,336	100.0

In the presented analyses and in multivariable regressions, categories 3 and 4 were collapsed into the category Historical sources/Works of art, and categories 5 and 6 were collapsed into Other/No data.

2 Faculties

Comparison across faculties was central to the analysis. Academic disciplines are neither well defined nor clearly distinct [1]. The same is true of faculties, i.e. clusters of disciplines like the

natural or social sciences. Still, many researchers have a clear understanding of which discipline and faculty they belong to. In this study, we relied on the participants own indication of their disciplinary home as the basis for comparison.

In the questionnaire, participants were asked, first, to select a general research area from a list of seven options reflecting common faculty divisions in European universities. The options were: “Natural science”, “Medical science”, “Social Science”, “Engineering” and “Law”, as well as a combined category called “Arts, humanities and theology” and “Other”. After choosing a general study area, participants identified their specific research area from a further list. The list was generated from the 2020 Danish BFI list [2], which groups all journals recognised as “scientific” by the Danish government into disciplines. The list distinguishes 64 distinct disciplines. To simplify it, these were grouped in accordance with methodological similarities. Thus, English, Spanish, Asia studies, etc. were grouped together into “language and area studies”. Similarly, the life sciences – biology, food science, agriculture, molecular biology, biotechnology, etc. – were grouped together. This resulted in a list of around 30 study areas (see S1). Table 2 shows the distribution of participants across faculties.

Table 2: Distribution of participants across their self-identified faculties.

**Faculty**	**Number of participants**	**Percentage**
Natural science and mathematics	224	16.6
Engineering	109	8.2
Science, Technology, Engineering, and Mathematics (STEM), total	333	24.8
Medical sciences	265	19.8
Social sciences	354	26.5
Law	61	4.6
Arts, Humanities and Theology (Humanities)	240	18.0
Other	83	6.2
**Total**	**1,336**	**100**

Most of those self-identifying their faculty as “Other” were included in the analyses of differences across faculties. To do this, we grouped their specific study areas into the five categories, defined in Table 2, according to where they are usually placed in major research universities (see Table 3 below). Study areas that either cut across faculties or were placed in different faculties in different universities were classified as “Other”.

Table 3: Grouping of the specific study areas of those who self-identified their general study area as “Other”.

Faculty	Specific study area	Number of participants
**STEM**	Life sciences (biology, biotech, molecular biology, agriculture, food sciences, sports science, etc.)	16
	Physics, chemistry, geoscience	2
	Mathematics, statistics and similar	2
	Computer science and similar	28
	Materials science, electrical engineering, chemical engineering, construction, transport and traffic or similar	2
	**Total STEM**	**50**
**Medical sciences**	Public health	3
	Nursing	1
	**Total Medical sciences**	**4**
**Social Sciences**	Economics	2
	**Total Social Sciences**	**2**
**Humanities**	Design (including architecture)	3
	History and art history	4
	**Total Humanities**	**7**
**Other**	Linguistics	3
	Pedagogy and didactics	9
	Psychology	4
	Interdisciplinary	4
	**Total Other**	**20**
**Total**		**83**

For the data analysis we then defined six broad faculties:

- **STEM**: Includes participants who self-identify as studying within the natural or engineering sciences *and* respondents who classify their general study area as “Other” and their specific study area as one of the STEM sciences listed in Table 3.

- **Medical sciences:** Includes participants who self-identify as studying within the medical sciences *and* respondents who classify their general study area as “Other” and their specific study area as one of the medical sciences listed in Table 3.
- **Social science**: Includes participants who self-identify as studying within the Social Sciences *and* respondents who classify their general study area as “Other” and their specific study area as one of the social sciences listed in Table 3.
- **Law**: Includes participants who self-identify as studying within Law
- **Humanities**: Includes participants who self-identify as studying within the general category “Arts, humanities and theology” *and* respondents who classify their general study area as “Other” and their specific study area to be one of the humanistic science listed in Table 3.
- **Other**: Includes participants who self-identify as studying within the general study area “Other” and also classify their specific study area as one of the study areas listed under “Other” in Table 3.

The 20 participants in the “Other” faculty were not included in the analyses of differences across faculties.

Finally, it should be noted that faculty classification also played an important role in the recruitment process. Here, we relied on a simpler clusters. The STEM and Medical sciences were grouped together, as were the social sciences and Law.

3: Disciplines represented in the sample

Table 4 shows the specific research fields indicated by participants after indicating their general field of research. A complete list of the research fields participants could select from is given in S1.

Table 4: Answers to the question "Please indicate the most relevant subcategory [of the general research field indicated in the previous question]".

Research field	Number of participants	Percentage
Anthropology ethnography, ethnology*	22	1.6
Archaeology	10	0.7
Bio, environmental and food engineering	18	1.3
Business	45	3.4
Cognitive science and psychology*	62	4.6
Computer science and similar*	76	5.7
Dentistry	9	0.7
Design (including architecture)*	9	0.7
Economics*	44	3.3
History and art history*	46	3.4
Human geography and developmental studies	7	0.5
Human medicine	121	9.1
Language and area studies	16	1.2
Law**	61	4.6
Life sciences (biology, biotech, molecular biology, agriculture, food sciences, sports science, etc.)*	137	10.3
Linguistics*	25	1.9
Literature, philosophy and history of ideas, drama, music, dance, culture and gender studies*	67	5.0
Materials science, electrical engineering, chemical engineering, construction, transport and traffic, or similar*	26	1.9
Mathematics, statistics and similar*	19	1.4
Media and communication science*	21	1.6
Medical technology	13	1.0
Nursing	4	0.3
Pedagogy and didactics*	45	3.4
Physics, chemistry, geoscience*	79	5.9
Political science	32	2.4
Public health*	49	3.7
Religious studies	4	0.3
Sports science*	7	0.5
Sociology*	47	3.5
Systems engineering	4	0.3
Theology	7	0.5
Veterinary medicine	12	0.9
Interdisciplinary*	93	7.0
Other*	99	7.4
Total	1,336	100

* Participants from multiple faculties chose this sub-category. The numbers have been merged.
**Participants indicating Law as their general research area were not asked to further specify.

References

[1] Morris SA, van der Veer Martens B. Mapping research specialties. Annual Review of Information Science and Technology, 2008;42(1):213-295.

[2] Ministry of Higher Education Denmark. The BFI lists. 2022. Available from https://ufm.dk/en/research-and-innovation/statistics-and-analyses/bibliometric-research-indicator/bfi-lists?set_language=en&cl=en
