# Supplementary material for: “The person in power told me to”—European PhD students’ perspectives on guest authorship and good authorship practice
Source: PLoS One. 2023 Jan 12;18(1):e0280018. doi: 10.1371/journal.pone.0280018 (PMC9836317; doi:10.1371/journal.pone.0280018)
Supplement: S4 File — (PDF) [file pone.0280018.s005.pdf]

S4: Details of recruitment

All participants were invited via emails distributed by their PhD school or PhD programme. Once a school or programme had agreed to participate, we provided a standard invitation, including a link to the online questionnaire, which they could distribute via their mailing lists. We encouraged our contacts to send out reminders, but not everyone did so.

When estimating response rates (Table 1 in the main text) we assumed that all PhD students in the relevant programmes were invited, and thus we equate the number of invited students with the publicly available, official number of students enrolled in the relevant PhD programmes.

1 Denmark

In Denmark, PhD students are enrolled in PhD schools, and most universities have roughly one PhD school per faculty. For recruitment, a complete list of the 152 PhD schools in Danish Universities was compiled and sorted according to the clusters defined in the main text. All 12 PhD schools in the social science, business and law cluster and 6 PhD schools in the humanities and theology cluster were contacted. Ten PhD schools from the STEM and medical sciences were drawn at random and invited as well. Central administrative personnel at the relevant PhD schools were contacted and were asked to help with recruitment.

Of the 28 schools contacted, 23 agreed to circulate the questionnaire (9 in STEM and medical science, 8 in social science, business and law, and 5 in humanities and theology). We prompted our contacts to send a reminder after about a week, but not everyone did so.

2 Hungary

In Hungary, PhD students in the 62 universities are organised in doctoral schools. The doctoral schools are not directly associated with the faculties, and there may be several doctoral schools organizing students within the same faculty. In the recruitment, 5 universities per cluster were

randomly selected. Then, all of the doctoral schools at the selected universities were listed, and 5 schools at each university offering doctoral programmes in the given faculty were randomly selected (if there were 5 or fewer, all were invited). A total of 92 doctoral schools were contacted (40 in STEM and medical science, 29 in social science, business and law, and 23 in humanities and theology), and 50 agreed to participate (22 in STEM and medical science, 17 in social science business and law, and 11 in humanities and theology).

3 Ireland

A total population recruitment was carried out in Ireland. All Deans of Postgraduate Studies, postgraduate student unions and student councils, as well as various other PhD networks within the nine Irish Universities were contacted. PhD schools from 7 universities, representing 82% of the total PhD population, agreed to participate.

4 Portugal

In Portugal, PhD students are organised in PhD programmes. Each faculty will normally have several such programmes. There are 702 programmes in total. In the recruitment, a complete list of programmes was compiled and sorted according to the clusters defined in the main text. Then, 294 programmes (65 in STEM and medical science, 87 in social science, business and law, and 142 in humanities and theology) from each cluster were drawn at random (in 3 rounds). 111 programmes agreed to participate (23 in STEM and medical science, 40 in social science, business and law, and 48 in humanities and theology).

5 Switzerland

In the French-speaking part of Switzerland, PhD students are organised in PhD programmes. Each faculty typically has several PhD programmes.

A total population recruitment was carried out in the French-speaking part of Switzerland. The rectorates of all 5 universities in the area (housing 30 faculties in total) were contacted and

asked to invite all PhD students in their university to engage with the study. Following this, 2 universities, with 12 faculties in total, agreed. The remaining universities did not respond. Subsequently, the deans of all of the relevant faculties in universities where no contact had yet been established were approached. An additional 7 faculties were recruited in this way. In the final stages of data collection, 2 specific programmes were selected by convenience and contacted in order to meet the inclusion criteria.
