# Supplementary material for: “The person in power told me to”—European PhD students’ perspectives on guest authorship and good authorship practice
Source: PLoS One. 2023 Jan 12;18(1):e0280018. doi: 10.1371/journal.pone.0280018 (PMC9836317; doi:10.1371/journal.pone.0280018)
Supplement: S5 File — (PDF) [file pone.0280018.s006.pdf]

S6: Descriptive statistics and average student for data analysis

Table 1. Descriptive statistics regarding all demographic and study-specific variables (n = 1336)

Variable	Category	Proportion
Gender identity	Male	0.38
	Female	0.56
	Other/prefer not to say	0.06
Country	Denmark	0.32
	Hungary	0.17
	Ireland	0.18
	Portugal	0.18
	Switzerland	0.15
Faculty	STEM	0.29
	Medical sciences	0.20
	Social sciences	0.27
	Humanities	0.18
	Law	0.05
	Other	0.01
Data type used	Quantitative	0.56
	Qualitative	0.20
	Historical/Works of art	0.13
	Other/No data	0.11
Age (years)		**M (SD)**
		32.49 (8.20)

Table 2. The average student used in the estimation of predicted probability to award guest authorship, and stated reasons for allowing, it in all study countries (n = 1096)

**Variable**	**Category**	**Proportion**
Gender identity	Male	0.39
	Female	0.55
	Other/prefer not to say	0.05
Country	Denmark	0.33
	Hungary	0.17
	Ireland	0.18
	Portugal	0.18
	Switzerland	0.13
Faculty	STEM	0.28
	Medical sciences	0.21
	Social sciences	0.27
	Humanities	0.19
	Law	0.05
Data type used	Quantitative	0.56
	Qualitative	0.19
	Historical/Works of art	0.13
	Other/No data	0.11
M Age (years)		**M (SD)**
		32.57 (8.20)
